# Dynamics of cell wall elasticity pattern shapes the cell during yeast mating morphogenesis

**DOI:** 10.1098/rsob.160136

**Published:** 2016-09-07

**Authors:** Björn Goldenbogen, Wolfgang Giese, Marie Hemmen, Jannis Uhlendorf, Andreas Herrmann, Edda Klipp

**Affiliations:** 1 Theoretical Biophysics, Institute of Biology, Humboldt-Universität zu Berlin, Invalidenstraße 42, 10115 Berlin, Germany; 2 Molecular Biophysics, Institute of Biology, Humboldt-Universität zu Berlin, Invalidenstraße 42, 10115 Berlin, Germany

**Keywords:** *Saccharomyces cerevisiae*, atomic force microscopy, cell wall elasticity, turgor pressure, tip growth, finite-element modelling

## Abstract

The cell wall defines cell shape and maintains integrity of fungi and plants. When exposed to mating pheromone, *Saccharomyces cerevisiae* grows a mating projection and alters in morphology from spherical to shmoo form. Although structural and compositional alterations of the cell wall accompany shape transitions, their impact on cell wall elasticity is unknown. In a combined theoretical and experimental approach using finite-element modelling and atomic force microscopy (AFM), we investigated the influence of spatially and temporally varying material properties on mating morphogenesis. Time-resolved elasticity maps of shmooing yeast acquired with AFM *in vivo* revealed distinct patterns, with soft material at the emerging mating projection and stiff material at the tip. The observed cell wall softening in the protrusion region is necessary for the formation of the characteristic shmoo shape, and results in wider and longer mating projections. The approach is generally applicable to tip-growing fungi and plants cells.

## 1. Introduction

The cell wall of fungi and plants maintains cell shape and structural integrity during morphogenesis, as well as in response to environmental challenges. Local compliance and global hydrostatic pressure are assumed to generate the shape of walled cells [1]. To accomplish asymmetric growth, cells need to generate spatially inhomogeneous wall properties by local synthesis and lysis of cell wall material. The model organism *Saccharomyces cerevisiae*, or yeast, offers a simple and well-studied system to investigate the impact of spatially altering physical cell wall properties on the morphogenesis of tip-growing cells. Here, we focus on yeast mating morphogenesis.

**Figure 1.**
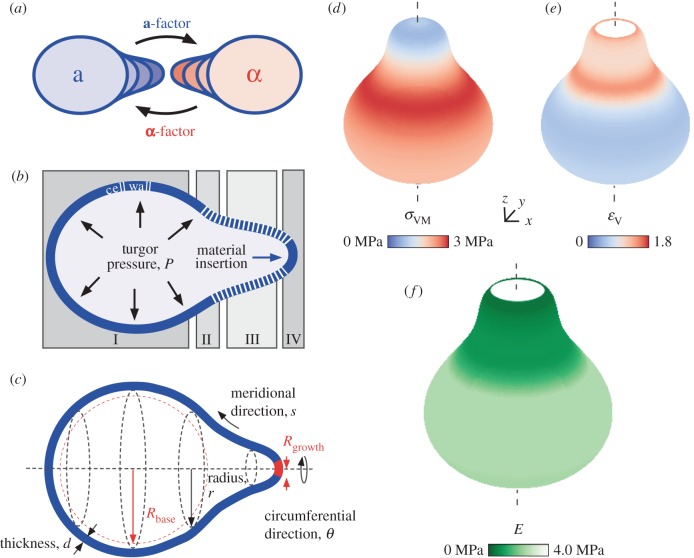
Elastic shell model of the shmoo predicts a spatially inhomogeneous distribution of the lateral cell wall stresses and the Young's modulus. (*a*) *MAT***a** cells synthesize **a**-factor and grow a mating projection towards *MAT***α** cells upon sensing **α**-factor, and vice versa. (*b*) Scheme of a shmooing cell divided in base (I), neck (II), shaft (III) and tip (IV) with considered contributing elements, i.e. elastic cell wall (blue) with locally varying Young's modulus (*white hatched*), turgor pressure *P,* and material insertion at the tip. (*c*) Description of coordinates given an axisymmetric geometry: circumferential angle *θ*, meridional length *s* with corresponding distance to the axis *r*, relaxed radii for the mating projection 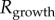 and the spherical part 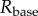 as well as cell wall thickness *d*. (*d*,*e*) Distribution of von Mises stress *σ*_VM_ and volumetric strain *ɛ*_V_, respectively, with red indicating high and blue low values. (*f*) Resulting spatial distribution of the Young's modulus *E*, dark green shows low values and shades of light-green to white high values. White area at the tip shows region of undefined *ɛ*_V_ and *E*. Contour plots of stress, strain and elastic modulus shown in electronic supplementary material, figure S2. We used a cell with relaxed radius of 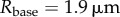, expanded radius of 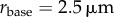, relaxed radius of 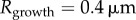, cell wall thickness of 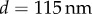, turgor pressure of 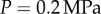 and Poisson's ratio of 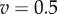. The values of stresses and the Young's modulus itemized by the base, neck, shaft and tip are shown in electronic supplementary material, table S1.

Yeast cells proliferate as single oval cells upon budding from a mother cell. Tip growth in yeasts occurs during pseudo-hyphal growth in response to nutrient depletion, as well as during mating. Haploid cells exist as either *MAT***a** or *MAT***α** mating type (figure 1*a*). To initiate mating, haploid cells either secrete mating pheromones **a**- or **α**-factor and are sensitive to the complementary pheromone [2–5]. During mating, two adjacent cells change shape significantly, each growing a mating projection in the direction of the pheromone gradient [6] to overcome the distance between the mating partners. In this way, the tip-growing cell, or shmoo, is essential for cell fusion and finally zygote formation.

During morphological changes, cell wall composition and structure must be flexible. The yeast cell wall is a strong and elastic two-layered structure and composed mainly of fibrillar β-1,3-glucan branched by β-1,6-linked glucose residues. The β-1,3-glucan fibrils serve as backbone and are linked to the more amorphous and higher branched β-1,6-glucan, as well as to the less abundant chitin and some mannoproteins [7]. Additionally, mannoproteins are linked through a processed glycosylphosphatidylinositol anchor to the β-1,6-glucan. In contrast to the inner layer, the outer layer is enriched in mannoproteins. However, cell wall structure and composition are not fixed, but subjected to alterations during growth or in response to environmental challenges [8].

Specifically, cell wall structure and, concomitantly, cell wall composition change during shmoo formation. In particular, the fraction of mannan in the cell wall declines, whereas glucan content rises [9], and the amount of 1,6-beta cross-links of the glucan network increases as well as the amount of chitin, which triples after pheromone induction [10]. Electron micrographs of shmooing cells revealed an altered cell wall structure at the protrusion side already at early stages of mating projection formation, and cells became more susceptible to lysis by glucanases [9]. Interestingly, electron micrographs showed only marginal variations of cell wall thickness, except for the very tip [11]. The reported changes in cell wall structure and composition probably lead to changes in mechanical properties of the cell wall.

The morphogenetic switch from isotropic growth to tip growth is highly regulated. Central elements of the pheromone pathway [12] and the cell wall integrity pathway in mating yeast (Rho1, PKC) were found to be located at the tip of the shmoo. Associated actin recruitment leads to an accumulation of vesicles loaded with cell wall precursors and remodelling enzymes at the tip of the mating projection [7,11] and, hence, focuses cell wall synthesis to the tip. Regulation of cell wall remodelling is crucial for cell integrity. For example, the absence of the cell wall integrity sensor Mid2 results in about 90% cell death after pheromone treatment [13].

Precisely how dynamic changes in cell wall composition and structure translate into alterations in shape remain unclear. To understand shape evolution during the shmooing process, it is necessary to account for both growth pattern variations and cell wall alterations, the latter of which lead to changes in mechanical properties of the cell wall. Thus far, theoretical studies on mating morphogenesis have focused on growth patterns and xneglected the influence of cell wall properties [14–16]. Nevertheless, several studies using computational models have evaluated the effects of varying material properties on tip growth of plant [17–21] and fungal cells [22–25], respectively. However, these concepts do not adequately describe the morphogenic switch from spherical to tip growth, as occurs during shmooing. Physical properties of the yeast cell wall have been studied [26,27], but spatial and temporal information on the mechanical cell wall properties relevant to mating morphogenesis is not yet available owing to technical limitations.

Recent advances in atomic force microscopy (AFM)-based multiparametric imaging, such as quantitative imaging (QI™), have significantly improved the resolution of local cell wall elasticity [8,28–34].

In this study, we began by formulating a steady-state model (SM) of yeast mating morphogenesis to rationalize the relevance of cell wall elasticity, turgor pressure and cell wall synthesis at the tip of the mating projection, for shmoo formation. To validate the obtained spatial cell wall elasticity distribution as a prerequisite for shmoo formation, we probed the surface of shmooing and non-shmooing *MAT***a** *bar1*Δ cells with AFM in QI™-mode and, thereby, searched for local variations in cell wall properties. Temporal and spatial elasticity distributions of living yeast cells provided insights into cell wall dynamics during mating projection formation. In light of these findings, we developed dynamic cell wall models (DMs) of mating morphogenesis, which describe dynamic changes in cell wall elasticity and potential influences on directed growth.

## 2. Results

### 2.1. Steady-state cell wall model

We constructed the SM based on continuum mechanics, accounting for turgor pressure, inhomogeneous material properties of the cell wall and local cell wall synthesis at the tip. To simplify mathematical description of the surface, we used an axisymmetric shape with a typical contour and size, observed with bright-field microscopy and described in the literature [4]. We distinguished between four cell wall regions based on the shape of the shmooing cell (figure 1*b*): base (I), neck (II), shaft (III) and tip (IV). The base region represents the spherically unaltered part of the cell, and the protrusion is described by two regions, the tip and the cylindrical part, the shaft. In addition, the transition between shaft and base was defined as the neck region.

The yeast cell wall can be considered as a thin pressurized shell—the inner surface of which is continuously acted upon by a constant turgor pressure—with a cell wall thickness that is small compared with cell diameter. In a spherical shell, the stress is uniformly distributed, but becomes inhomogeneous if the shape deviates from a sphere (electronic supplementary material, figure S1). To calculate the spatial distribution of the plane stresses in the cell wall of a shmooing cell, we assumed an axisymmetric geometry as depicted in figure 1*c* (electronic supplementary material, figure S2*a*) and a constant cell wall thickness *d* as well as a constant turgor pressure *P*. Each position on the shell is characterized by the circumferential angle *θ* and the meridional distance *s*, where *s* is zero at the tip. The radius *r* is a function of *s* and describes the distance from the axis of symmetry. From the principal stresses, circumferential stress *σ_θ_* and meridional stress *σ*_S_, we calculated the von Mises stress *σ*_VM_. Compared with the base, *σ*_VM_ was reduced at the shaft and increased at the neck (figure 1*d* and electronic supplementary material, figure S2*b*,*c*). At the tip, stress highly depended on the curvature of the tip, e.g. a flat tip led to high stresses at this tip. To obtain information on the spatial distribution of material properties of a shmoo-shaped shell we used linear constitutive relationships between stresses and strains for plane elasticity [35]:2.1
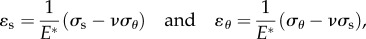
where 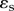 and 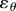 are meridional and circumferential strains, respectively. 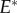 is the Young's modulus for plane elasticity and 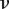 is Poisson's ratio. The Young's modulus (or elastic modulus) *E* is related to 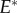 by 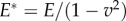 and is a measure of material elasticity, whereby higher *E*-values correspond to stiffer material and low *E*-values to softer material.

We assumed that cell wall material in the mating projection originates from a circular region at the tip, whereas the cell wall fraction in the spherical region originates from earlier isotropic cell wall synthesis. Thus, we presumed a relaxed shape, i.e. for zero turgor pressure (see osmotic stress experiments below), of a shmooing cell with two distinct radii defining the shape, 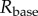 and 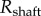 (electronic supplementary material, figure S2*a*). Because intracellular vesicles were found to be directed to the tip during the shmooing process [11], we considered material insertion and cell wall synthesis to occur only at the tip of the mating projection. Hence, the assumption of pure elastic material at the tip must fail and the tip was consequently excluded from strain and elasticity calculations.

From the spatial stress and strain distributions, we derived the spatial distribution of *E* (figure 1*f* and electronic supplementary material, figure S2*b*), required for maintaining cell shape in steady state. Owing to lower stresses and larger strains in the protrusion region, *E* is reduced at the shaft compared with *E* at the base. Variation of *R*_shaft_ and resulting strain and *E-*profiles are shown in the electronic supplementary material, figure S2*e*,*f*. Increasing *R*_shaft_ resulted in lower strain values and hence higher *E*-values at the shaft, however qualitative *E*-pattern remained similar (electronic supplementary material, figure S2*e*,*f*). Notably, assuming constant volumetric strain, the *E*-profile followed the stress profile and, hence, resulted also in lower *E*-values at the shaft (electronic supplementary material, figure S2*g*,*h*). In summary, under the assumption of (i) elastic deformation of isotropic material, (ii) constant turgor pressure, (iii) a confined region of growth at the tip, and (iv) a given geometry (the shmoo), the elasticity of the cell wall must spatially differ to maintain the shape of the shmoo.

### 2.2. Cell wall nano-indentation experiments

**Figure 2.**
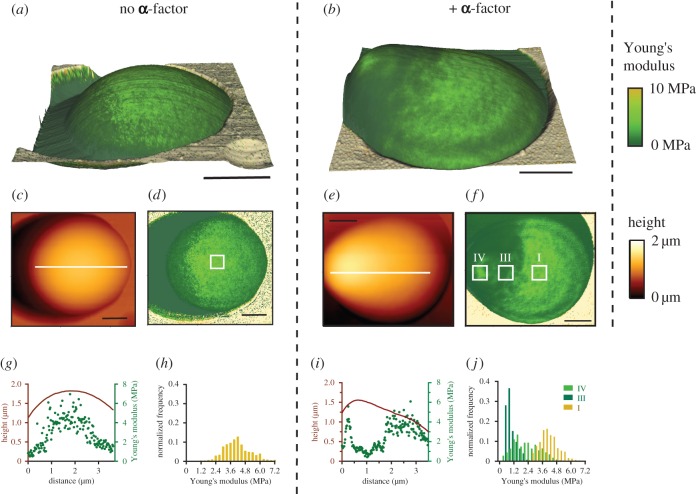
**α**-factor treatment of *MAT***a** *bar1*Δ cells induced a localized softening of the cell wall in the region of the emerging mating projection. (*a*,*b*) Three-dimensional images with textures representing the elasticity for two individual *MAT***a** *bar1*Δ cells without (*a*) and with (*b*) 1 h treatment with 10 µM **α**-factor; black scale bars, 1 µm. (*c*,*e*) Height images and (*d*,*f*) Young's modulus maps of cells shown in (*a*) and (*b*); black scale bars, 1 µm. Note that the colour scale for the elasticity ranges from 0 to 10 MPa to distinguish the cell from its stiffer surrounding (orange spectrum). (*g*,*i*) Cross sections of height (red curve) and Young's modulus (green dots) maps at the indicated white lines in (*c*,*e*). Young's modulus histograms for regions marked with white frames in (*d,f*) are shown in (*h,j*) with *n* = 625 and *n* = 225, respectively. The three marked frames correspond to three of the four identified characteristic regions (figure 1*b*) of the induced mating projection: I the base, III the shaft and IV the tip. Note, that an analysis of a comparable frame at the neck (II) would not be appropriate owing to the strong gradient of the Young's modulus at the neck, as can be seen in (*i*).

To experimentally investigate spatial alterations of the *E*-distribution along the cell wall during shmooing and, thereby, to check the predictions of the SM, we probed the surface of *Saccharomyces cerevisiae* with AFM (electronic supplementary material, figure S3). Using the advanced force spectroscopy-based AFM QI™ method [29], we acquired information on height and elasticity at each scanning position, and generated height and *E* maps (figure 2*c*–*f*) at nanoscale resolution. Both data types were acquired simultaneously, which could be visualized in combination as colour-coded three-dimensional representations (figure 2*a,b*). Here, *z*-values refer to height, whereas colour represents elasticity of the yeast cell.

For indentation experiments of pressurized shells, at least two different regimes exist, depending on indentation depth. At large indentations, the whole shell deforms and the restoring force is mainly defined by the turgor pressure [36], whereas at small indentations with sharp indenters, only the shell material is deformed and force is governed by material properties of the shell [37]. Because we focused on elastic properties of the cell wall, we used AFM cantilevers with sharp pyramidal tip geometry and limited applied force to 1.5 nN. The measure of applied force corresponded to indentation depths of approximately 40 nm at the base of shmooing cells (electronic supplementary material, figure S4), which are significantly smaller than the reported cell wall thickness of 115 nm [38].

To study elasticity changes during shmooing, we used cells of *MAT***a** mating type in the late exponential phase with and without induction by the mating pheromone **α**-factor. All experiments were conducted in sterile filtrated synthetic drop-out medium at 22°C, allowing cells to metabolize and proliferate during measurements. Individual living yeast cells were trapped in a porous polycarbonate membrane with a nominal diameter of 3 or 5 µm [39]. To ensure successful induction with **α**-factor, we used the *MAT***a** *bar1*Δ strain lacking the pheromone-specific protease Bar1 [40]. Actual measurements were performed as follows: first, we scanned fields of 40 × 40 µm at low resolution (64 × 64 pixels) to localize suitable trapped cells. Subsequently, we scanned smaller areas of 4 × 4 µm–8 × 8 µm enclosing the target cell at a higher resolution (256 × 256 pixels or 128 × 128 pixels). To temporally resolve the formation of the mating projection, settings were optimized for high scanning speed. To compare local *E-*values of the cell wall between cells, or between regions within a cell, flat regions from 0.25 to 0.4 µm^2^ at the top of the cells were analysed. As an added benefit, this protocol avoided possible artefacts that could arise from probing the surface of spherical objects. Artificially low *E*-values arise if the tip of the cantilever slides over the strongly tilted surface instead of indenting the shell, e.g. at the edge of the accessible half-sphere [41].

The cell wall of untreated *MAT***a** *bar1*Δ cells exhibited a spatially uniformly distributed elasticity *E* (figure 2*a*,*d*,*g* and electronic supplementary material, figure S5) with the exception of the aforementioned artificial reduction at the edges. In agreement with previous reports [27,42], we observed locally altered *E*-values at sides of previous budding events, so-called bud scars (electronic supplementary material, figure S3). By comparing *E-*values of regions at the top of trapped cells (figure 2*d*), but outside of possible bud scars, we obtained *E*-values of 2.67 ± 0.35 MPa (mean ± s.e.m., *n* = 12) for the cell wall of untreated *MAT***a** *bar1*Δ cells.

When *MAT***a** *bar1*Δ cells were incubated with a high concentration of **α**-factor (10 µM) for more than 90 min, we observed typical changes in morphology, i.e. protrusion of the mating projection, by AFM (figure 2*b*,*e*,*i*). Intriguingly, we also found local variations in *E-*values of the cell wall, which were not observed for untreated cells. By combining height and elasticity maps in three-dimensional images (figure 2*b*), where texture represents the local Young's modulus, we found that the decrease in *E* covered the whole protrusion region, except for the tip. To systematically analyse this observation, we compared the mean *E* of the base (I), the shaft (III) and the tip (IV), as shown in figure 2*f*,*j* (electronic supplementary material, figure S5), for all cells exhibiting a mating projection. By eye, we manually selected these regions (I, III, IV) based on topographic maps and knowledge of shmoo shape derived from microscopy images, while ensuring minimal height gradients in these regions. Although, *E*-values (mean ± s.e.m.) varied strongly between different cells (electronic supplementary material, figure S6), the decrease in *E* between the base (2.53 ± 0.50 MPa, *n* = 7) and the shaft (0.69 ± 0.22 MPa, *n* = 7), and between the tip (1.8 ± 0.72 MPa, *n* = 7) and the shaft of an individual cell was found to be significant (non-parametric Friedman test, *p* < 0.002) (electronic supplementary material, figure S6). In contrast, *E-*values of the tip and the base did not differ significantly. Because *E*-values varied strongly between cells, we also plotted mean values of *E*_shaft_, *E*_tip_ and *E*_base_ against corresponding mean values of *E*_base_ for every cell to analyse the degree of reduction in *E* from base to shaft.

Height and elasticity profiles provided a visualization of the Young's modulus gradient at the neck. We used these profiles to test whether variations of the Young's modulus could be explained by variations in height profile. To compare the shape of the height profile with the *E-*profile, we chose equatorial cross sections of the observed cell (figure 2*g*,*i*). For non-treated cells (figure 2*g*), the *E-*profile roughly followed the height profile. *E* was decreased at the edges and increased on the top of the cells, most probably owing to sliding effects on tilted surfaces as mentioned earlier. Cross sections further revealed a high point-to-point variance of *E* at the top, which is also reflected in the broad distribution of *E* exemplarily shown in figure 2*h*.

In contrast, the *E*-profiles of shmooing cells did not follow the height profile, although we observed decreased *E-*values at the edges (figure 2*i*). The observed Young's modulus dropped locally rather abruptly to about one-fifth of former *E*-values at the neck, assuming the neck of the protrusion to be at the location where the height profile starts to differ from the circular or ellipsoid shape. At the very same position, we observed an abrupt drop of *E*, without a change of slope in the height profile. *E*-values remained low with the exception of the presumed tip of the protrusion, where values rose again. Note that the tip does not necessarily have to be located at the highest peak, as its location depends on the orientation of the shmooing cell in the pore. Therefore, the observed decrease in the Young's modulus at the neck cannot be explained by variation of the height profile in that region.

**Figure 3.**
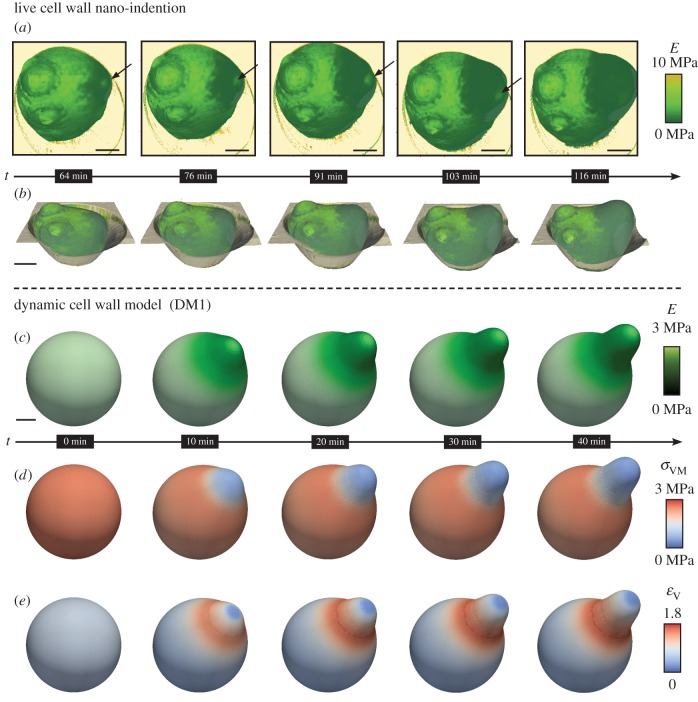
Dynamics of cell wall elasticity during shmooing, measured by live cell wall nano-indentation experiments and simulations of the dynamic cell wall model (DM1). Softening of the cell wall starts early, continues with elongation of the mating projection and forms a ring around its base. *MAT***a** *bar1*Δ cells were induced with 10 µM **α**-factor for 64 min before the first image was acquired. (*a*,*b*) Sequences of height and elasticity developments during formation of a mating projection, from a continuous AFM measurement ((*a*) consecutive images of the *E-*distribution and (*b*) three-dimensional reconstruction with elasticity pattern, see electronic supplementary material, movie S1). Arrows indicate a region of non-softened cell wall material at the tip; scale bar, 1 µm. Elasticity pattern controls shape of the evolving shmoo in the dynamic model. (*c*–*e*) Simulation snapshots of DM1 (electronic supplementary material, movie S2), showing the pattern of the Young's modulus ((*c*), black/green/white), the von Mises stress 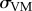 ((*d*), blue/red) and the volumetric strain 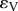 ((*e*), blue/red); scale bar, 1 µm*.* Note that colour scale for the elasticity in (*a*) ranges from 0 to 10 MPa in order to distinguish the cell from its stiffer surrounding (orange spectrum). Contour plots of stress, strain and elastic modulus are shown in electronic supplementary material, figure S11.

Motivated by these observations, we further investigated the dynamics of the morphogenetic switch, with a focus on the initiation of the mating projection. In doing so, intriguing questions arose regarding the stage of morphogenesis at which cell wall softening takes place and how this influences the evolving shape. Therefore, we traced the shape and elasticity of single *MAT***a** *bar1*Δ cells (*n* = 3) over time, from the early stage protrusion to the late stage elongated mating projection (figure 3*a* and electronic supplementary material, movie S1 and figure S7). Cells were continuously scanned, resulting in a complete image every 12–15 min. Remarkably, the region of the initial reduction of cell wall elasticity denoted the origin of the subsequently formed mating projection. Note that owing to the aforementioned apparent decrease of the Young's modulus on tilted surfaces [41], the uncertainty of elasticity measurements increases with increasing length of the mating projection. The softening of the cell wall continued during the formation of the mating projection; however, *E*-values did not decrease in the remaining (adjacent) round-shaped region. Furthermore, the boundary between high and low *E-*values at the transition from the unaltered cell to the mating protrusion became less frayed and more circular with time. The region at the tip of the evolving mating projection, indicated with arrows in figure 3*a*, and the white frame IV in figure 2*f*, remained stiffer than the surrounding area. Moreover, we found defined rigid areas even at early stages of mating projection development.

Because reported values for turgor pressure ranged from 0.1 to 1.0 MPa [43–46], we estimated the turgor pressure for our experiments using the cellular stiffness *k* (electronic supplementary material, figure S8) upon large cell indentations [47]. In this case, turgor pressure can be approximated using the formula [36]2.2
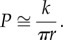


The obtained turgor pressure of 0.21 ± 0.05 MPa (mean ± s.e.m., *n* = 3) was in the reported range. Furthermore, this turgor pressure resulted in a relaxed radius of 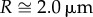, i.e. the unstressed cell wall enclosed 50% of the volume of the pressurized cell (electronic supplementary material, figure S9).

In conclusion, nano-indentation measurements confirmed the extensive decrease of the Young's modulus at the shaft of an existing mating projection as predicted by the SM. Intriguingly, we observed a spot of unaltered elasticity at the tip of the emerging mating projection that was not covered by the SM.

### 2.3. Dynamic cell wall models

Inspired by the AFM data on dynamics of cell wall elasticity during yeast mating morphogenesis, we investigated the impact of the obtained elasticity pattern on growth dynamics during shmooing. Simulation of growth dynamics clearly goes beyond the steady-state model, and thus required development of dynamic and more advanced cell wall models to predict shape evolution.

In the dynamic models (DMs), the cell wall was implemented using a triangular surface mesh, which is commonly used for finite-element simulations. The triangular elements were subjected to plane stresses arising from constant turgor pressure, which continuously acts on each surface element. Each triangular element was allowed to deform in an either elastic [48] or elastoplastic manner corresponding to the acting forces (electronic supplementary material, figure S10). Spatial inhomogeneities were introduced by assigning individual material properties to each triangular element. In this manner, the DMs could cover locally varying cell wall elasticity as well as cell wall thickness.

**Figure 4.**
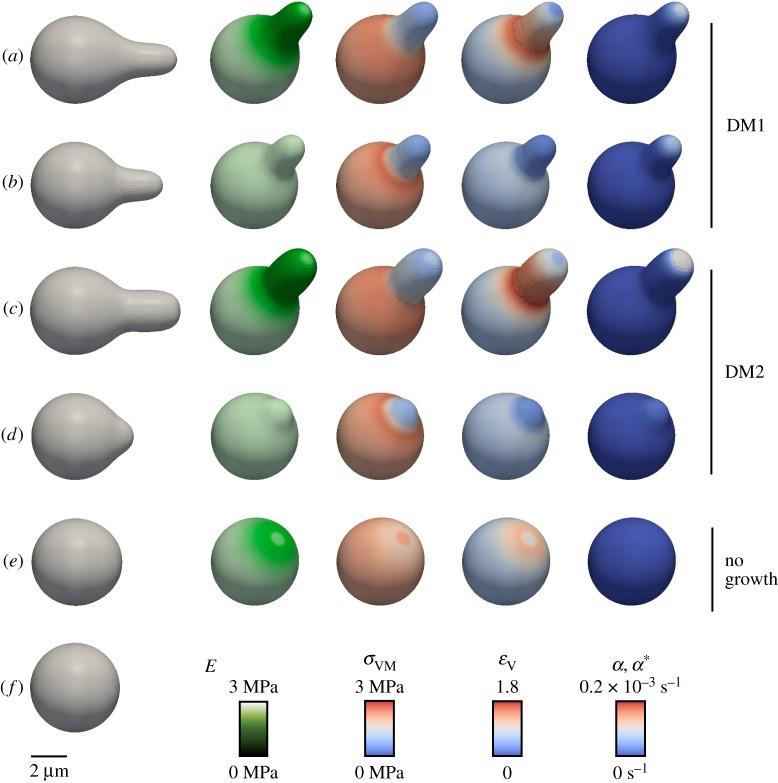
The influence of elasticity patterns on cell wall growth for two variants of the dynamic cell wall model, DM1 and DM2. Snapshots of the dynamic cell wall model DM1 (electronic supplementary material, movie S2) and DM2 (electronic supplementary material, movie S3) after 1 h of simulated time, where the shape (column 1), the Young's modulus *E*, the von Mises stress 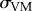, the volumetric strain 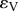 and expansion rates 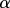 or 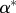 is shown for different model assumption; scale bar, 2 µm. (*a*,*b*) Simulations of DM1 assuming growth under yield stress. (*c*,*d*) Simulations of DM2 assuming growth under yield strain. (*e*) Simulation of pure elastic expansion without cell wall growth. (*f*) Spherical reference shape. The elasticity pattern (*a*,*c*) results in a longer mating projection than for homogeneous elasticity (*b*,*d*). The effect of elasticity pattern on cell growth is even larger for DM2 (*c*) than for DM1 (*a*). Growth showed a ring-like pattern around the tip for the DM2 ((*c*), column 5), whereas for the DM1 ((*a*), column 5) growth was focused to the centre of the tip. Contour plots of stress, strain and elastic modulus for DM1 and DM2 are shown in electronic supplementary material, figure S11.

The simulations (figure 3*c*–*e* and figure 4; electronic supplementary material, movies S2 and S3) started from a pressurized sphere with relaxed radius *R*_base_ = 2.0 µm, turgor pressure of *P* = 0.2 MPa, Poisson's ratio 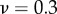 and homogeneous elasticity *E* = 2.5 MP as obtained for untreated cells, resulting in a radius of 2.5 µm for the expanded shell. In agreement with the experimental data and to reflect the dynamics of the softer region, *E*-values were set to 0.7 MPa in a circular region at the tip (regions II and III), whereas *E*-values in the centre of that region with a radius of 0.4 µm (region IV) were set to 1.8 MPa. In agreement with our temporal data, the region of decreased *E*-values extended during dynamic simulation with 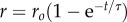, where *r* was the distance to the centre of the reduction and *τ* was arbitrarily set to 200 s. The reduction in *E*, together with the turgor pressure, led to an expansion of the elastic wall and thus to an asymmetric shape (figure 3*c*–*e* and figure 4; electronic supplementary material, figure S11, movies S2 and S3).

We calculated the local plane von Mises stresses 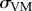 for the shell [49]. In these calculations, we used the second Piola–Kirchhoff stress tensor, which represents the plane forces per area (see electronic supplementary material, text S1 for derivation). The spatial 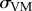 distribution obtained with the DMs under the assumption of shmoo geometry and inhomogeneous elasticity was consistent with the stress pattern obtained with the SM (electronic supplementary material, figures S2*b* and S11*b*). The observed stress patterns showed three distinct characteristics: a diffuse belt of enhanced stress at the neck, generally reduced stress at the rest of the protrusion and slightly increased stress at the tip. Thus, as long as a given cell shape is considered, all models—SM and the DMs—are comparable. Moreover, stress patterns are entirely controlled by geometry at steady state (i.e. no or very slow growth forces at equilibrium). Higher *E*-values at the tip led to a less curved tip, which in turn resulted in higher stress values in that region.

We calculated the local volumetric plane strain 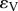 for each triangular mesh element (electronic supplementary material, text S1). The strain pattern followed the stress pattern when the elasticity was homogeneously distributed (figure 4*b*,*d*). However, inhomogeneous distribution of elasticity resulted in both patterns becoming incongruous (electronic supplementary material, figure S11*b*,*c*), because local strain increases with lower *E*-values. In contrast to the stress pattern, we observed a belt of high strain at the neck, slightly shifted towards the shaft, and a decrease in strain at the tip.

To test different hypotheses on cell growth, we implemented two DMs. In the dynamic model 1 (DM1), we modelled cell growth as plastic cell wall expansion upon a yield limit following the concept of Lockhart [50]. Because only the nascent fungal cell wall is supposed to expand plastically [51,52], we implemented yielding only at the tip of the mating projection. Inspired by the bell-shaped distribution of polarization markers [53] during pheromone response, we defined extensibility 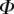 as2.3
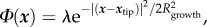
where 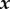 is the position on the cell wall, 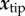 is the position of the tip, 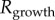 defines the extension of the growth zone and 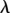 is stress-dependent expansion velocity. The expansion rate is given by2.4
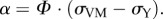


As mentioned above, strain and stress patterns are significantly different owing to an inhomogeneous elasticity distribution. Therefore, we implemented a second dynamic model (DM2), where we alternatively tested strain-dependent yielding [19,54]. The expansion rate becomes2.5
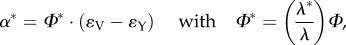
where 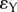 is the yield strain and 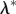 is strain-dependent expansion velocity. Both expansion rates 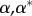 have the same unit (s^−1^). Taking into account the observed elasticity pattern of both growth models, the stress-driven DM1 and the strain-driven DM2, we could reproduce the characteristic shmoo shape (figure 4*a*,*c* and electronic supplementary material, figure S11). Furthermore, in both models, the elasticity pattern changed the geometry of the base from spherical to rather egg-shaped. To assess the effect of the elasticity pattern, we simulated stress- and strain-dependent growth with homogeneous elasticity as well (figure 4*b*,*d*). Using the realistic elasticity pattern led to significantly longer and wider mating projections compared to growth with homogeneous elasticity (compare figure 4*a* with *b* and *c* with *d*). The shape of the shmoo remained unaltered for different 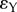 or 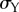 (electronic supplementary material, figure S12), although the expansion rate increased for lower yield limits. Therefore, we chose the parameters 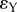, 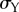, 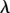 and 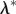 such that the expansion rate during shmoo formation was comparable (electronic supplementary material, table S1) to growth with elasticity pattern (figure 4*a*,*c*), leading coincidentally to differences in shape for growth with homogeneous elasticity (figure 4*b*,*d*). However, stress- and strain-dependent scenarios showed significant differences in tip geometry. While stress-dependent (DM1) expansion was focused to the tip and resulted in a more pointed tip, strain-dependent (DM2) expansion was increased in a ring around the stiffer region and resulted in a flatter tip (figure 4*a*,*c* and electronic supplementary material, figure S11*a*). As a third alternative, we tested pure elastic expansion without yielding (figure 4*e*), resulting in a rather egg-like shape instead of a shmoo.

**Figure 5.**
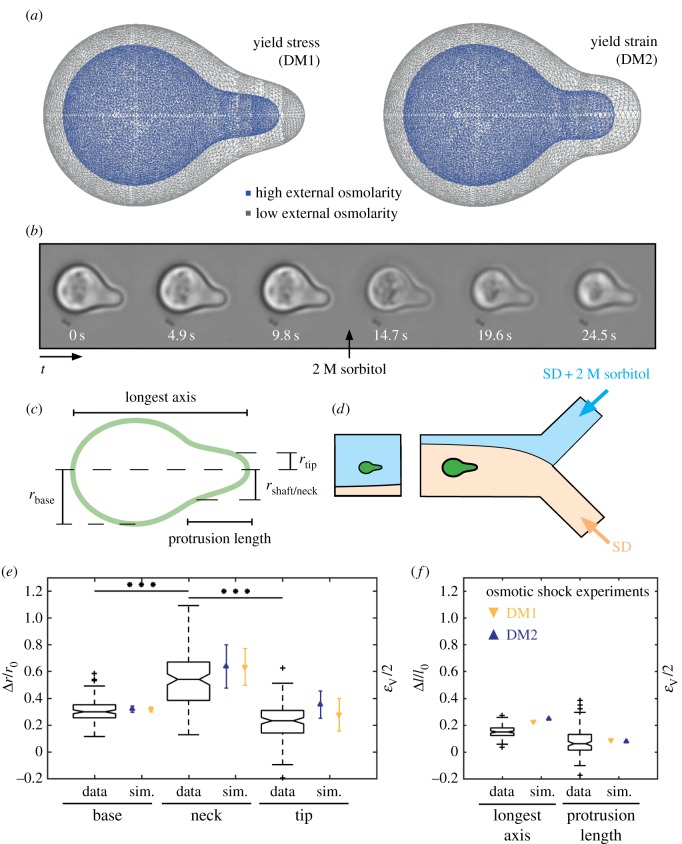
Osmotic stress experiments confirmed strain profiles of the dynamic models. (*a*) Modelled shapes under yield stress (left) and yield strain assumption, before (grey) and after (blue) reduction of turgor pressure, respectively. (*b*) Time series of bright-field microscopy images of shmooing *MAT***a** *bar1*Δ cells rapidly exposed to high extracellular osmolyte concentration (2 M sorbitol) in a microfluidic device (MFD). (*c*) Scheme of a shmooing cell with indicated dimensions: length of protrusion and longest axis, radii of base, neck and tip. (*d*) Scheme of the MFD set-up. The interface between normal (SD) and high osmolarity (SD + 2 M sorbitol) media can be shifted by varying the input pressures, which allows to rapidly exchange the cellular environment. (*e*,*f*) Measured relative expansion Δ*r*/*r*_0_ and Δ*l*/*l*_0_ in black and simulated volumetric strain obtained from yield stress model (DM1) as yellow triangles or from yield strain model (DM2) as blue triangles, for base, neck and tip, respectively. The deformation Δ*r*/*r*_0_ (*e*) at the neck was significantly higher than at the base or at the tip (*t*-test, ***p < 0.001, *n* = 119), whereas longest axis and protrusion show only a slight relative expansion Δ*l*/*l*_0_ (*f*).

To challenge the models and their predictions on the cell wall strains (figures 4 and 5*a*), we estimated the deformation of shmooing yeast cells upon severe osmotic shock, using a reported protocol [23]. With 2 M sorbitol, we used medium with high osmolarity to estimate the relaxed cell shape under approximately zero turgor pressure. At osmotic conditions with osmolarity greater than 1.15 osmole kg^−1^, plasmolysis occurs in yeast cells [55,56], indicating that the cell wall detaches from the plasma membrane, such that the observed shape of the cell wall is the relaxed cell shape. Furthermore, we rapidly exchanged the external medium, using a microfluidic system (figure 5*d*), to avoid adaptation of the cells to the high external osmolyte concentration and, thus, to monitor only the elastic response of the cell wall. We followed the shape change of single cells (*n* = 119) with time series of bright-field microscopy images (figure 5*b*), which we manually analysed. The time between the switch from low to high external osmolarity and the measurements of strain was in the range of 25–35 s. For both states, low and high turgor pressure, we defined five characteristic dimensions (figure 5*c*): the radii 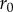 and 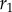 of the base, the neck and the tip as well as the length 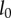 and 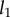 of the protrusion and the longest axis, respectively. For all dimensions, we calculated the relative expansion 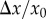 and compared it with the predicted half volumetric strain 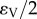
from both models, i.e. DM1 and DM2 (electronic supplementary material, figure S11). Note that the half volumetric strain is identical to 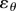 only for spherical geometry, at the neck the 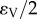 is a lower estimate for 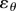.

Essentially, the computationally estimated strains were consistent with the measured strains with regard to the absolute values and the local profile (figure 5*e*,*f*). The strain increased significantly from the base to the neck and decreased again at the tip, although the increase at the neck was less and the drop at the tip was more pronounced than predicted. The measured circumferential strain (mean ± s.d., *n* = 119) was 0.30 ± 0.08, 0.55 ± 0.21, 0.23 ± 0.14 for base, neck and tip, respectively, whereas the predicted 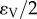 values were 0.32 ± 0.02, 0.64 ± 0.14, 0.26 ± 0.12 for the DM1 and 0.32 ± 0.02, 0.64 ± 0.16, 0.35 ± 0.10 for the DM2, respectively.

In contrast, the longest axis deformed only slightly (0.15 ± 0.04) and the protrusion length showed nearly no change (0.08 ± 0.09) during the osmotic shock experiments.

Estimations of the Young's modulus from the measured strains are generally difficult, because the local stresses highly depend on the actual geometry of the cell, as we pointed out for the SM. However, the Young's modulus at the base could be calculated if this part was assumed to be a sphere (see supporting electronic supplementary material, text S1). From the measured strain of the base, we calculated a Young's modulus *E* of 2.58 ± 0.8 MPa (mean ± s.d., *n* = 119), assuming a wall thickness of 115 nm and a turgor pressure of 0.2 MPa.

## 3. Discussion

We reported the temporal and spatial distribution of cell wall elasticity during mating morphogenesis of *S. cerevisiae.* In particular, we focused on the switch from isotropic growth to directed growth of a mating projection. In a combined theoretical and experimental approach, we showed that cell wall softening in the protrusion region is necessary to obtain the characteristic shmoo shape. Nano-indentation experiments on the cell wall of living shmooing cells, performed with AFM in QI™ mode, provided us with height and elasticity maps, from which we reconstructed the local elasticity of the accessible yeast cell wall. AFM measurements revealed a characteristic spatial distribution of cell wall elasticity at the emerging protrusion, with a zone of softer cell wall material surrounding a centre of unaltered stiffer cell wall material. For the first time, we could describe spatial and temporal changes in mechanical cell wall properties accompanying the switch from spherical to tip growth. We implemented two variants of a dynamic cell wall model, DM1 and DM2, in a finite-element framework, which considered the cell wall mainly as an elastic pressurized shell with plastoelastic material in the growth region. By integrating the experimentally derived spatial elasticity distribution, the model was capable of generating the characteristic shmoo shape. Computational assessment of model variants revealed that the characteristic cell wall elasticity pattern enables shmooing cells to grow farther than with homogeneous elasticity.

### 3.1. Nano-indentation on the yeast cell wall

In agreement with earlier studies, we showed that cell wall elasticity of non-stimulated yeast cells is homogeneously distributed, with the exception of potential bud scars [8,57]. For pheromone-treated cells, experiments led to three main observations.

First, cell wall elasticity remained homogeneous and constant for the non-protruding part of the yeast cell. Using AFM, we observed mean (±s.d.) local Young's moduli at the base of **α**-factor-stimulated cells and non-stimulated cells of 2.53 ± 1.30 and 2.67 ± 1.20 MPa, respectively, which were consistent with the calculated value of 2.58 ± 0.8 MPa from the osmotic shock experiments. This agreement between values of the modulus estimated from AFM and from osmotic treatment suggests that mechanical anisotropy of the yeast cell wall is negligible. Resulting *E*-values were in the range of earlier reported *E*-values of 1.62 ± 0.22 MPa acquired with AFM [58], but differed by two orders of magnitude from *E*-values acquired with whole cell compression experiments [59].

Second, and in agreement with the SM, our AFM experiments revealed an extensive decrease of the local Young's modulus at the shaft. Interestingly, the reduction of cell wall stiffness started at very early stages of the shmooing process. In fact, reduction of the local Young's modulus was the first indication of an emerging protrusion in AFM measurements. An extensive local decrease in the Young's modulus of the yeast cell wall has not yet been reported.

Other tip-growing walled cells, such as plant pollen tubes, have shown a decrease in cellular stiffness during tube elongation [20,60]. The elasticity profile of plant pollen tubes differed significantly from yeast, because the cellular stiffness in plant pollen tubes dropped gradually from the shaft to the tip. The cellular stiffness might not directly translate into the local Young's modulus. Intriguingly, simulations of multicellular plant tissues predicted a varying elasticity between epidermal cells at the site of morphogenic changes [54]. Cells in the protrusion region were supposed to be softer than in the remaining tissue. Reduced stiffness at the initial growth site appears to be a common growth strategy of walled cells from single cells to whole tissues.

Third, we observed a spot of stiffer cell wall material in the region of softer cell wall material at the presumed tip. We found this area to be most prominent at early stages of the shape transition. Although increased stiffness at the tip of growing cells has been discussed in theory [17], such a spot has not yet been measured. Micro-indentation experiments on pollen tubes have shown ambiguous results on the development of stiffness towards the tip, both increasing and decreasing in stiffness [60–62], whereas the uppermost tip was not accessed in the reported experimental set-ups.

### 3.2. Reasons for alterations of mechanical properties at the tip

Green [63] argued that the velocity of vesicle transport might influence stiffness at regions of rapid growth. Vesicles transport cell wall material as well as lytic enzymes to the plasma membrane. Thus, depending on the time vesicles need to reach the surface, the lytic enzymes will loosen the transported cell wall material differently. Because vesicles reach the polarization site faster owing to transport along the actin cables, the cell wall material is supposed to be stiffer at the region of rapid growth compared with more distant regions of the tip.

### 3.3. Cell wall model predictions and implications

The successive construction of the SM and the DMs allowed us to assess the role of mechanical cell wall properties in shmoo formation during yeast pheromone response. In the SM, geometry and resulting stresses and strains determined the elasticity distribution, whereas in the DMs, the experimentally determined time-resolved elasticity distribution governed shape evolution. In all models, a diffuse belt of enhanced stresses was predicted around the neck, depending on elasticity at the shaft. High tensile stress values at the neck may reach the critical failure stress of the cell wall [26], and might be one reason for increased cell death during mating. Coping with enhanced stress values at the neck of protrusions is challenging not only for single walled cells but also for budding plant tissues, as has been demonstrated by numerical simulations of the shoot apical meristem [54]. Additionally, both DM1 and DM2 predicted a belt of strongly increased volumetric strain at the shaft and the neck compared to the base and the tip. Osmotic shock experiments showed that both the profiles and the values of the strain were consistent with the prediction from DM1 and DM2, although the predicted circumferential strain at the neck was a lower estimate and might be twice as high. Reported chitin deposition at the neck might limit the high strains at the neck to avoid critical values and prevent bursting [64].

### 3.4. Strain-driven versus stress-driven growth

Cell growth is commonly described as plastic, hence irreversible, cell wall expansion limited by a yield threshold. Here, we compared and tested two different yield criteria: yield stress (DM1) and yield strain (DM2). In principle, both criteria are equivalent if material elasticity is homogeneous, but produce different results if elasticity varies locally. Based on the observed inhomogeneous elasticity distribution, we tested for a possible influence of the elasticity pattern on plastic expansion, i.e. growth process. Following the classical Lockhart-like yield stress criterion, yield stress is significantly smaller during tip elongation than during spherical expansion, because stresses in the protrusion are reduced compared with those in the base. Hence, the cell requires a mechanosensor that is dedicated to the shmoo formation, generates a reduced yield stress and localizes to the tip. The cell wall integrity sensor Mid2 might play such a role, since Mid2 has been shown to be crucial for survival during mating, and is localized to the shmoo tip [13,65].

The highest stresses in the presumed growth zone were found at the spot of unaltered *E*-values, resulting in a focused growth at this spot and rather pointed tip geometry if the yield stress criterion (DM1) was applied. This is in agreement with reports on the localization at the tip of Ccw12, a mannoprotein that is thought to maintain structural integrity at the site of actual growth [66]. In contrast, applying a yield strain criterion (DM2) instead of a yield stress criterion (DM1) led to a circular growth zone around the tip and blunter tip geometry, because the spot of stiffer material reduced strain at the very tip. Furthermore, the strain was generally higher in the mating projection than in the base. Therefore, reduced elasticity facilitates cell wall expansion under the yield strain criterion. In contrast to the yield stress criterion, cell wall expansion during spherical and tip growth could be obtained with the same yield limit if a yield strain criterion was applied, although focusing of the growth to the tip is still required.

### 3.5. Model assessment

Growth and shmooing are complex processes whereby available data allow a certain degree of freedom in the formulation of an appropriate model. We strived for a simple description based on qualitative shape changes of shmooing yeasts and on quantitative data obtained from AFM measurements. In the following, we discuss possible alternatives.

Theoretically, similar cell shapes could be obtained by either gradual isotropic expansion or by uniform anisotropic expansion [63,67]. However, no evidence of anisotropic cell wall expansion has been reported for *S. cerevisiae*.

The product of 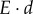 (thickness) is an invariant of the elastic shell models, e.g. decrease in *E* can be compensated by an increase in *d*. The invariance of 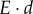 implies that a locally varying cell wall thickness could equally modulate cell shape, as recently shown for leaf trichome morphogenesis [21]. In shmooing yeast, a stiffer cell wall may be required to counteract cell wall thinning at the tip. To assess and compare our results, some aspects need to be considered. We assumed the thickness of the cell wall to be constant, which is mainly consistent with EM micrographs [9,11], except for reduced cell wall thickness at the very tip in some cases.

The cell wall was considered in both models as a single-layered thin shell, but the yeast cell wall consists of two distinct layers: an electron-dense outer layer enriched in mannoproteins and an electron-transparent polysaccharide layer. The incorporation of an elastic double-layer model [68] may be worth considering for a more comprehensive model. While the model described the relation between elasticity patterns and shape changes very well, the establishment and regulation of elasticity patterns reported herein remain unclear. Indeed, an important and challenging question is how pheromone signalling and its localization [14,15,69,70] control the development of local cell wall elasticity and extensibility. During the yeast pheromone response, cell wall synthesis and cell wall remodelling machinery are recruited to the polarization site. Of particular interest are the confinement of cell wall softening to the protrusion region, the exclusion of the tip, and the definition of growth zones.

The benefit of the presented approach is not limited to *S. cerevisiae*, but generally applicable to other walled cells, such as fungi and plants cells, to test hypotheses on morphogenesis. In particular, our finite-element model is based on refinement and plastic growth and, therefore, allows for large shape changes without remeshing. For instance, the pathogenic fungus *Candida albicans* invades tissues by forming hyphae [71]. An understanding of this morphogenic switch via a possible local change in cell wall elasticity might help to develop specified drugs against the progression of this pathogen. In addition, adaptation to cell wall anisotropy of the presented approach, combining the AFM and finite-element modelling, would increase the application range from bacterial cells to plant cells [72].

## 4. Conclusion

In summary, we integrated detailed spatio-temporal AFM data into a spatio-temporal shell model of the cell wall. Our combined theoretical and experimental approach enabled us, for the first time, to evaluate the impact of local shell elasticity on shape and stress evolution during mating morphogenesis of *S. cerevisiae*. We showed that the cell wall softens in the protrusion region and that this softening is necessary to obtain the characteristic shmoo shape. Furthermore, we observed a spot of stiffer material at the tip of the emerging mating projection. In our cell wall model, the stiffer spot at the tip led to different growth patterns during strain- or stress-dependent cell wall expansion.

## 5. Material and methods

### 5.1. Steady-state model

The SM was based on a simple constitutive relationship for linear elasticity and plane stress. It assumed axisymmetric cell shape, where every point on the cell wall can be expressed in terms of the meridional distance *s* measured from the end of the base and the circumferential angle *θ*. This description allowed expressing stresses and strains in terms of the local principal curvatures. The principal stresses were given by the meridional stress 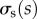 and the circumferential stress 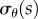 (electronic supplementary material, figure S2*c*) [35]:5.1
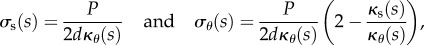
where 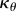 and 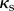 are the principal curvatures at each given position of the shell. For plane stress the von Mises stress 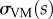 is given by5.2
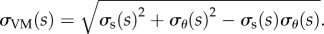


Here, 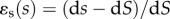 and 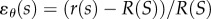 are the meridional and circumferential strains, respectively. While d*S* denotes a small relaxed and d*s* the actual extend of a small surface patch in meridional direction, *S* is the meridional distance of the relaxed shape measured from the base end. Using the relationship [73]5.3
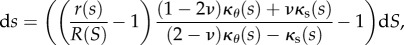
points of the relaxed and natural shape could be identified starting from the end of the base. Therefore, strains and stresses were calculated from the parameters *P*, *d* and 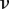 the geometry of the relaxed and natural shape only. Note that the strains were not calculated in the growth region, because we assume plastic growth at the shmoo tip. The plane elasticity 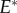 corresponding to the given shapes was computed from the linear constitutive relationship [35]5.4
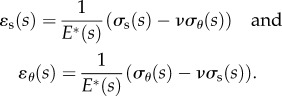


### 5.2. Yeast cell culturing

Wild-type *MAT***a** strains and the deletion strain *bar1*Δ used in this study were based on BY4741 (*MAT***a** *his3Δ1 leu2Δ0 met15Δ0 ura3Δ0*) [74]. Cell cultures were grown at 30°C in sterile-filtered synthetic drop-out medium (SD; 0.17% yeast nitrogen base without amino acids, 0.5% ammonium sulfate, 2% glucose, 55 mg l^−1^ adenine, 55 mg l^−1^ l-tyrosine, 55 mg l^−1^ uracil, 20 mg l^−1^ l-arginine, 10 mg l^−1^ l-histidine, 60 mg l^−1^ l-isoleucine, 60 mg l^−1^ l-leucine, 40 mg l^−1^ l-lysine, 10 mg l^−1^ l-methionine, 60 mg l^−1^ phenylalanine, 50 mg l^−1^ l-threonine and 40 mg l^−1^ l-tryptophan) overnight to a late exponential or early stationary phase.

### 5.3. Cell trapping and pheromone induction

Yeast cells were mechanically immobilized as previously described [39]. Briefly, cell cultures were shortly shaken to separate the cells and reduce cell aggregates. About 10 ml of the cell suspension was subsequently squeezed through a polycarbonate filter (Whatmann, GE Healthcare Europe GmbH, Freiburg, Germany) with a nominal pore diameter of 5 or 3 µm, respectively. The filter paper was gently rinsed with sterile filtrated SD medium to remove attached non-trapped cells and prevent contamination with unsolved particles in the solution. Subsequently, the polycarbonate membrane was glued rapidly on a cleaned glass surface of a fluid chamber using addition-vulcanizing silicone Alpa-Sil V 66 (Alpina-Technische Produkte GmbH, Geretsried, Germany). After a 5 min attachment period, the filter was rinsed again with SD and mounted on the AFM sample holder. Bright-field microscopy was used to control for free cells and occupancy of the filter holes. For shmoo formation, cells were induced with 10 µM **α**-factor (Sigma Aldrich, St Louis, MO).

### 5.4. Live cell wall nano-indentation

AFM overview scans, 64 × 64 pixels, were performed in QI™-mode to localize individual trapped cells, which were neither embedded too deeply nor extended too far above the surrounding surface. Selected cells were subsequently scanned in higher spatial resolution (256 × 256 pixels).

All nano-indentation measurements were performed with an AFM (Nanowizard III, JPK, Berlin, Germany) mounted on an inverted optical microscope (Axio Observer.Z1 Carl Zeiss AG, Oberkochen, Germany) at room temperature in SD medium, using a triangular-shaped cantilever MLCT-E/F (Bruker, Camarillo, CA) with a nominal spring constant of 100 and 500 mN m^−1^, respectively. The actual spring constant was determined using the thermal noise calibration method [75]. For overview as well as high-resolution scanning, the force spectroscopy-based imaging QI™ mode (electronic supplementary material, figure S3) was applied, such that applied force never exceeded 1.5 nN. The approach and retraction speed were not identical and chosen such that scanning time was minimized. To obtain the Young's modulus (*E*) of the cell wall the extend curves for each pixel, corrected for the tip sample separation, were fitted to a Hertz–Sneddon model [76] assuming a conical indenter with half-angle of 18° and a Poisson's ratio of 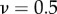. The analysis of AFM images and force–distance curves was performed by JPK data processing software (JPK, Berlin, Germany).

To maximize scanning speed, it was essential to maximize approach velocity (approx. 70 µm s^−1^), and thus indentation velocity, without a distinct change in *E* (electronic supplementary material, figure S13). This may have led to a drop in accuracy of the determined *E* and therefore may partially explain the discrepancy in *E*-values of untreated cells from 2.62 ± 1.03 MPa to earlier reported *E*-values from 1.62 ± 0.22 MPa (mean ± s.d.) [58].

However, the high scanning speed (approx. 3.5 s per line) was necessary not only to gain a picture of temporal changes in cell wall elasticity but also to obtain height and elasticity map of shmooing cells in high resolution (256 × 256). High-resolution maps were required for the identification of spatial elasticity patterns and their comparison to the accessible topography of the cell.

Note, we evaluated elasticity at the tip of the mating projection by calculating the mean Young's modulus from a quadratic region of about 0.25 µm^2^ at the presumed location of that tip (figure 2*f*). However, boundaries of chosen frames occasionally exceeded the region of increased *E*-values at the tip and may have led to a reduced mean Young's modulus.

### 5.5. Dynamic models

The DMs are based on continuum mechanics and are described in detail in the electronic supplementary material. The models were implemented in the general purpose finite-element toolbox DUNE [77]. The cell wall is represented as an unstructured triangular surface mesh, which is commonly used for finite-element simulations. A spherical surface mesh was constructed using the finite-element mesh generator Gmsh [78]. The triangular elements were assumed to deform elastoplastically. Below a given yield limit, these elements were modelled as elastic biquadratic springs [48] based on the plane stress assumption. Above the yield limit, the relaxed edges of these triangles were elongated by an expansion rate depending on the applied yield criteria. Two different yield criteria were implemented: (i) yield stress and (ii) yield strain. The extensibility was confined to the tip by assuming a bell-shaped distribution with mean at the tip. Each triangular element was allowed for different material properties, which enabled us to model inhomogeneous material properties of elasticity, growth and cell wall thickness. The expansion rate was small compared with the elastic forces such that the elastic forces during growth were close to equilibrium. Triangular elements exceeding a threshold on the edge length were decomposed by regular refinement into four triangular elements. The mesh interface used allows for regular refinement and hanging nodes, which naturally occur in the case of two neighbouring elements with a different level of refinement. The local refinement enabled the simulation of the cell wall growth for large deformations without remeshing of the surface grid.

### 5.6. Wall strain assessment

The deformation state of the cell wall of shmooing *MAT***a** *bar1*Δ cells was assessed by evaluating the cell shape before and after exposure to high osmotic medium, using a microfluidic device [23]. The yeast cells were grown to an OD between 0.3 and 0.6 in SD medium and subsequently induced with 10 µM **α**-factor. After 2 h, cells were seeded into a Y-shaped microfluidic device (MFD). To avoid wash-out during measurement, cells were covalently attached to the glass surface of the MFD (figure 5*d*) using concanavalin A (Sigma Aldrich). Cell shape was monitored with an IX83 microscope (Olympus Deutschland GmbH, Hamburg, Germany) using a 100× oil immersive objective (Plan S Apo 1.4 NA, Olympus) and a back-illuminated EM-CCD camera (iXON Ultra 888). During the acquisition of z-stack time series, we rapidly changed the extracellular medium from SD to SD with 2 M sorbitol (Sigma Aldrich) using a fluid pressure controller (Fluigent GmbH, Jena, Germany). To ensure constant pheromone induction, we added 10 µM **α**-factor to both media. Shape was assessed using extended focal images from the Cell Sense Dimensions software (Olympus Deutschland GmbH, Hamburg, Germany).

### 5.7. Statistical analysis

Statistical analyses were performed using GraphPad Prism (GraphPad Software, La Jolla, CA) and MATLAB and Statistics Toolbox Release 2015b (The MathWorks, Inc., Natick, MA).

## Supplementary Material

Supplementary Figures and Table
