# Supplementary material for: Dynamics of cell wall elasticity pattern shapes the cell during yeast mating morphogenesis
Source: Open Biol. 2016 Sep 7;6(9):160136. doi: 10.1098/rsob.160136 (PMC5043577; doi:10.1098/rsob.160136)
Supplement: Supplementary Figures and Table [file rsob160136supp1.pdf]

Dynamics of cell wall elasticity pattern shapes the cell during yeast mating morphogenesis.

Björn Goldenbogen[1], Wolfgang Giese[1], Marie Hemmen[1], Jannis Uhlendorf[1], Andreas Herrmann[2], Edda Klipp[1]

1. Institute of Biology, Theoretical Biophysics, Humboldt-Universität zu Berlin, Invalidenstraße 42, 10115 Berlin
2. Institute of Biology, Molecular Biophysics, Humboldt-Universität zu Berlin, Invalidenstraße 42, 10115 Berlin

Content

Supporting Text S1 1
Dynamic Cell Wall Models (DM) 1
Steady State Cell Wall Model (SM) 6
References 9
Figures S1 - S13 10
Table S1 Parameters values used in the models 24

Supporting Text S1

Dynamic Cell Wall Models (DM)

We developed a computational model to simulate inhomogeneous elasticity and elasto-plastic growth due to internal turgor pressure in space and time. The approach is based on continuum mechanics, where plane stress for thin shells is assumed. The cell wall is discretized into a triangular mesh using the finite element mesh generator Gmsh (Geuzaine and Remacle 2009) and simulations are performed with the general purpose finite element framework DUNE (Bastian et al. 2008). We simulated the model equations in different situations for a wide range of parameters. Furthermore, the analytical results derived for the steady state model (SM) were compared and matched with the dynamic models DM1 and DM2.

First, we introduce the elastic properties of a single triangle in the discretization of the cell wall as suggested by Delingette (Delingette 2008). Let T be a triangle of the cell wall discretization, where the points of the undeformed triangle are denoted by P_1, P_2, P_3 and the points of the elastically deformed triangle $\hat{T}$ are denoted by Q_1, Q_2, Q_3, as depicted in Fig. S10. In the remainder, we refer to the elastically undeformed and deformed also as relaxed and stretched triangle, respectively. The area of the relaxed and stretched triangle is denoted as A_T and $\hat{A}_T$, respectively. The evolution of the cell wall was described by a deformation function $\Phi(\vec{x}, t)$. The right Cauchy-Green deformation tensor was computed from

$$C = \nabla\Phi^T \nabla\Phi \tag{1}$$

and the Green-Lagrange strain tensor from

$$\mathcal{E} = \frac{1}{2}(C - I). \tag{2}$$

These tensors were used to calculate the elastic energy, elastic forces and yield criteria. There are three important quantities that were expressed in terms of angles and length of the sides of the deformed and the relaxed triangle (Delingette 2008):

$$\mathrm{tr}(C) = \frac{1}{A_T}\left(l_1^2 \cos\alpha_{T,1} + \ l_2^2 \cos\alpha_{T,2} + \ l_3^2 \cos\alpha_{T,3}\ \right), \tag{3}$$

$$\mathrm{tr}(\mathcal{E}) = \frac{1}{A_T}\Big((l_1^2 - L_1^2)\cos\alpha_{T,1} + \ (l_2^2 - L_2^2)\cos\alpha_{T,2} + (l_3^2 - L_3^2)\cos\alpha_{T,3}\ \Big), \tag{4}$$

$$\det(C) = \frac{\hat{A}_T^2}{A_T^2}. \tag{5}$$

Here, $L_{T,i}$ and $l_{T,i}$ are the lengths and A_T and $\hat{A}_T$ are areas of the relaxed triangle and stretched triangle respectively. The angles of the relaxed triangle are denoted by $\alpha_{T,i}$. A sketch of the relaxed state and stretched triangle can be found in Fig. S10. The elastic energy of a single triangle *T* with thickness *d* is given by

$$W_T^{\mathrm{el}} = \frac{\lambda}{2}\mathrm{tr}(\mathcal{E})^2 + \frac{\mu}{2}\ \mathrm{tr}(\mathcal{E}^2)$$

$$= \sum_{i=1}^{3} \frac{k_{T,i}}{4}\left(l_{T,i}^2 - L_{T,i}^2\right)^2 + \sum_{i \neq j}^{3} \frac{c_{T,i}}{2}\left(l_{T,i}^2 - L_{T,i}^2\right)^2\left(l_{T,j}^2 - L_{T,j}^2\right)^2. \tag{6}$$

The coefficients depend on the Young's modulus E^* for plane elasticity, the cell wall thickness d and the Poisson's ratio ν. The Young's modulus for plane elasticity is related to the 3D Young's modulus *E* by

$$E^* = \frac{E}{(1-\nu^2)}. \tag{7}$$

Here, μ and λ are the Lamé parameters, which are given by

$$\lambda = \frac{E^*\nu}{(1-\nu^2)}. \tag{8}$$

$$\mu = \frac{E^*}{(1+\nu)}. \tag{9}$$

The coefficient $k_{T,i}$ can be interpreted as tensile stiffness and the coefficient $c_{T,i}$ as angular stiffness. These coefficients read

$$k_{T,i} = E^* d \frac{2cot^2\alpha_i + 1 - \nu}{16(1-\nu^2)A_T}, \tag{10}$$

$$c_{T,i} = E^* d \frac{2cot\ \alpha_{T,i} cot\ \alpha_{T,j} - 1 + \nu}{16(1-\nu^2)A_T}. \tag{11}$$

From the elastic energy of each triangle the resulting forces at vertex i were computed as

$$\vec{F}_{T,i}^{\mathrm{el}} = \sum_{i \neq j} k_{T,j} \left(l_{T,k}^2 - L_{T,k}^2\right)\left(\vec{Q}_{T,j} - \vec{Q}_{T,i}\right) + \sum_{i \neq j} \left(c_{T,j}\left(l_{T,i}^2 - L_{T,i}^2\right) + c_{T,i}\left(l_{T,j}^2 - L_{T,j}^2\right)\right)\left(\vec{Q}_{T,j} - \vec{Q}_{T,i}\right). \tag{12}$$

In this representation the coordinates of the stretched triangle are represented by $\vec{Q}_{T,i}$. The total force at each vertex was computed from the sum of the forces induced by the turgor pressure and the counteracting elastic force:

$$\vec{F}_{T,i} = \vec{F}_{T,i}^{\mathrm{turgor}} - \vec{F}_{T,i}^{\mathrm{el}}. \tag{13}$$

The forces induced by the turgor pressure were computed from:

$$\vec{F}_{T,i}^{\text{turgor}} = \frac{1}{3} A_T P \vec{n}_T. \tag{14}$$

The equation of motion for every vertex of the mesh reads

$$\ddot{\vec{x}}_n = \sum_{\substack{T \\ \text{with } x_n \in T}} \frac{1}{m_n} \vec{F}_{T,i}. \tag{15}$$

The mass m_i was computed from the triangle area as $m_n = \frac{1}{3}\rho A d$, where *A* is the area of all triangles that share the vertex *n*.

Elasto-plastic growth and yield criteria

The elasto-plastic growth depends on a yield stress criterion. In case the value calculated from the yield criterion was above a given yield limit, new material was inserted. In case the yield limit was not reached, we assumed only elastic deformations (see figure S10). Computationally this process was modeled by an irreversible deformation of the relaxed triangle. For these plastic deformations, we tested two yield criteria, yield stress σ_y (model DM1) and yield strain ε_y (model DM2). As yield stress criterion we used the von Mises yield criterion for plane stress (Yu 2006):

$$\sigma_{\text{Y}} < \sigma_{\text{VM}} = \sqrt{\sigma_1^2 + \sigma_2^2 - \sigma_1 \sigma_2} \quad , \tag{16}$$

where σ_1 and σ_2 are the principal stresses. Assuming a spherical shmoo tip with typical radius $0.5\ \mu m - 1.0\ \mu m$ and a turgor pressure of 0.2 MPa the maximum and von Mises stress is approximately 0.5MPa to 1.0 MPa. It shall be noted, that in case of a cylinder the Maximum stress differs from the von Mises stress:

$$\sigma_{\max} = \frac{Pr}{d}, \qquad \sigma_{\text{VM}} = \frac{3Pr}{4d}. \tag{17}$$

For the dynamic model the stress criterion was computed from the linear stress tensor:

$$S = \lambda\,\mathrm{tr}(\mathcal{E})I + 2\mu\mathcal{E}. \tag{18}$$

The trace of *S* is given by

$$\mathrm{tr}(S) = 2\lambda\,\mathrm{tr}(\mathcal{E}) + 2\mu\,\mathrm{tr}(\mathcal{E}), \tag{19}$$

and the determinant by

$$\det(S) = (\lambda^2 + 2\lambda\mu)\mathrm{tr}(\mathcal{E})^2 + \mu(\det(C) - \mathrm{tr}(C) - 1). \tag{20}$$

Using these quantities, the von Mises stress criterion can be computed as follows

$$\sigma_{\mathrm{VM}} = \sqrt{\mathrm{tr}(S)^2 - 3\det(S)}. \tag{21}$$

The subsequent expansion rate was calculated from

$$\alpha = \begin{cases} \Phi \cdot (\sigma_{\mathrm{VM}} - \sigma_{\mathrm{Y}}) \text{ if } \sigma_{\mathrm{VM}} > \sigma_{\mathrm{Y}} \\ 0, \quad \text{else.} \end{cases} \tag{22}$$

For the extensibility Φ a bell-shaped distribution around the tip was assumed

$$\Phi(\vec{x}) = \lambda e^{-\frac{|\vec{x}-\vec{x}_{\mathrm{Tip}}|^2}{2R_{Growth}^2}}. \tag{23}$$

The expansion of the cell wall was modeled as elongation of the edges of the relaxed triangle. Let T and T' be two adjacent triangles and $L_{T,T'}$ the relaxed length of the edge that both triangles share. The expansion rates α_T and $\alpha_{T'}$ were calculated as above for the triangles T and T', respectively. The elongation of the edge $L_{T,T'}$ is given by

$$\frac{1}{L_{T,T'}}\frac{dL_{T,T'}}{dt} = \frac{1}{2}(\alpha_T + \alpha_{T'}). \tag{24}$$

For the DM2 we modelled cell wall expansion upon a yield strain. Here, we used the volumetric strain as a measure for yielding:

$$\varepsilon_V = \mathrm{tr}(\mathcal{E}). \tag{25}$$

The extensibility is in this case given by $\Phi^* = (\lambda^*/\lambda)\,\Phi$. Which leads to the expansion rate

$$\alpha^* = \begin{cases} \Phi^* \cdot (\varepsilon_{\mathrm{V}} - \varepsilon_Y) \; if \; \varepsilon_{\mathrm{V}} > \varepsilon_{\mathrm{Y}} \\ 0, \quad else. \end{cases} \tag{26}$$

The elongation of the edge $L_{T,T'}$ is then analogously given by

$$\frac{1}{L_{T,T'}}\frac{dL_{T,T'}}{dt} = \frac{1}{2}\left(\alpha_T^* + \alpha_{T'}^*\right). \tag{27}$$

Steady State Cell Wall Model (SM)

In a mechanistic model for the cell shape, it is crucial to describe the distribution of forces due to turgor pressure, material insertion and the counterbalancing forces, which can be derived from the material properties of the cell wall. The first basic relationship connects the stresses (force per unit area) on the cell wall to the turgor pressure P, the cell wall thickness d and the local geometry. The latter is characterized by the principal curvatures. We assumed the cell shape to have a rotational symmetry and the shape of the cell was described as a surface of rotation. The distribution of the corresponding stresses are expressed in terms of curvatures (Flügge 1973):

$$\sigma_s(s) = \frac{P}{2d\kappa_\theta(s)}, \qquad \sigma_\theta(s) = \frac{P}{2d\kappa_\theta(s)}\left(2 - \frac{\kappa_s(s)}{\kappa_\theta(s)}\right). \tag{28}$$

Here, the principal curvatures are given by the meridional curvature, κ_s, and the circumferential curvature, κ_θ. The stresses were connected to the strains with a constitutive relationship for linear elasticity (Flügge 1973)

$$\begin{pmatrix}\sigma_s(s)\\ \sigma_\theta(s)\end{pmatrix} = \frac{E^*(s)}{(1-\nu^2)}\begin{pmatrix}1 & \nu\\ \nu & 1\end{pmatrix}\begin{pmatrix}\varepsilon_s(s)\\ \varepsilon_\theta(s)\end{pmatrix}, \tag{29}$$

where $E^*(s)$ is the Young's modulus for plane elasticity,ν is the Poisson's ratio, $\varepsilon_s(s)$ and $\varepsilon_\theta(s)$ are the meridional and circumferential strain, respectively. The Young's modulus and strains are functions depending on the arc length s (figure 1, figure S1, figure S2).

Here, $\varepsilon_s(s) = \frac{ds-dS}{dS}$ and $\varepsilon_\theta(s) = \frac{r(s)-R(s)}{R(s)}$ are the meridional and circumferential strain, respectively. While dS denotes a small relaxed and ds the actual extend of a small surface patch in meridional direction, S is the meridional distance of the relaxed shape measured from the base end. As for the dynamic model, the von Mises stress and volumetric strain are given by:

$$\sigma_{\mathrm{VM}}(s) = \sqrt{\sigma_s^2(s) + \sigma_\theta^2(s) - \sigma_s(s)\sigma_\theta(s)}, \tag{30}$$

$$\varepsilon_{\mathrm{V}}(s) = \varepsilon_{\mathrm{s}}(s) + \varepsilon_{\theta}(s)\,. \tag{31}$$

From equations (28) and (29) we can derive a relationship between circumferential and meridional strain, which only depends on the geometry and the Poisson's ratio

$$\varepsilon_s(s) = \varepsilon_\theta(s)\frac{(1-2\nu)\kappa_\theta(s) + \nu\kappa_s(s)}{(2-\nu)\kappa_\theta(s) - \kappa_s(s)}. \tag{32}$$

Using this relationship, we get the fomula

$$ds = \left(\left(\frac{r(s)}{R(S)} - 1\right)\frac{(1-2\nu)\kappa_\theta(s)+\nu\kappa_s(s)}{(2-\nu)\kappa_\theta(s)-\kappa_s(s)} - 1\right)dS, \tag{33}$$

(see also (Bernal, Rojas, and Dumais 2007)) which was used to identify points of the relaxed and natural shape of the cell starting from the end of the base. Therefore, strains and stresses are calculated from the parameters P, d and ν and the geometry of the relaxed and natural shape only. Note, that the strains are not calculated in the growth region, since we assume plastic growth at the shmoo tip. Inserting this relationship in the constitutive model we get:

$$\varepsilon_\theta(s) = \frac{r(s)-R(s)}{R(s)} = \frac{1}{E^*(s)}\left(\frac{P}{2d\kappa_\theta(s)}\left(2 - \frac{\kappa_s(s)}{\kappa_\theta(s)}\right) - \nu\frac{P}{2d\kappa_\theta(s)}\right). \tag{34}$$

For a given cellular geometry, the elasticity distribution was computed from (Bernal, Rojas, and Dumais 2007):

$$E^*(s) = \frac{R(s)}{r(s)-R(s)}\left(\frac{P}{2d\kappa_\theta(s)}\left(2 - \frac{\kappa_s(s)}{\kappa_\theta(s)}\right) - \nu\frac{P}{2d\kappa_\theta(s)}\right). \tag{35}$$

We calculated the stress and elasticity distribution for the shmoo shape shown in Fig. 1 (see main text) and Fig. S2. For the numerical computations, cubic spline functions were used to characterize the shape. The *NumPy* and *SciPy* package in Python was used for this purpose. Calculations for different assumptions on relaxed and extended cell shape and strains are shown in Fig. S2.

In the special case of a sphere or a cylinder, the strains, the stresses and the elasticity can be computed analytically (see Fig. S1). For a sphere, the circumferential and meridional strain are given by

$$\varepsilon_s = \varepsilon_\theta = \frac{r-R}{R} = \frac{1-\nu}{E^*}\frac{Pr}{2d}, \tag{36}$$

and the principal stresses are given by

$$\sigma_s = \sigma_\theta = \frac{Pr}{2d}. \tag{37}$$

Assuming a spherical geometry at the base as well as tip, the Young's modulus the tip can be approximated by the following formula:

$$E^* = (1-\nu)\frac{PrR}{2d(r-R)}. \tag{38}$$

This formula was used to compare the Young's Modulus obtained from AFM measurements and the Young's modulus estimated by osmotic shock experiments.

References

Bastian, P, M Blatt, A Dedner, C Engwer, R Kloefkorn, R Kornhuber, M Ohlberger, and O Sander. 2008. “A Generic Grid Interface for Parallel and Adaptive Scientific Computing. Part II: Implementation and Tests in DUNE.” *Computing* 82 (2-3): 121–38. doi:10.1007/s00607-008-0004-9.

Bernal, Roberto, Enrique Rojas, and Jacques Dumais. 2007. “The Mechanics of Tip Growth Morphogenesis: What We Have Learned From Rubber Balloons.” *Journal of Mechanics of Materials and Structures* 2 (6): 1157–68. doi:10.2140/jomms.2007.2.1157.

Delingette, Herve. 2008. “Triangular Springs for Modeling Nonlinear Membranes.” *Ieee Transactions on Visualization and Computer Graphics* 14 (2): 329–41. doi:10.1109/TVCG.2007.70431.

Flügge, Wilhelm. 1973. *Stresses in Shells*. Second. Springer-Verlag, New York-Heidelberg. doi:10.1007/978-3-642-88291-3.

Geuzaine, Christophe, and Jean-Francois Remacle. 2009. “Gmsh: a 3-D Finite Element Mesh Generator with Built-in Pre- and Post-Processing Facilities.” *International Journal for Numerical Methods in Engineering* 79 (11): 1309–31. doi:10.1002/nme.2579.

Yu, Mao-Hong. 2006. *Generalized Plasticity*. Springer Science & Business Media.

Figures S1 - S13

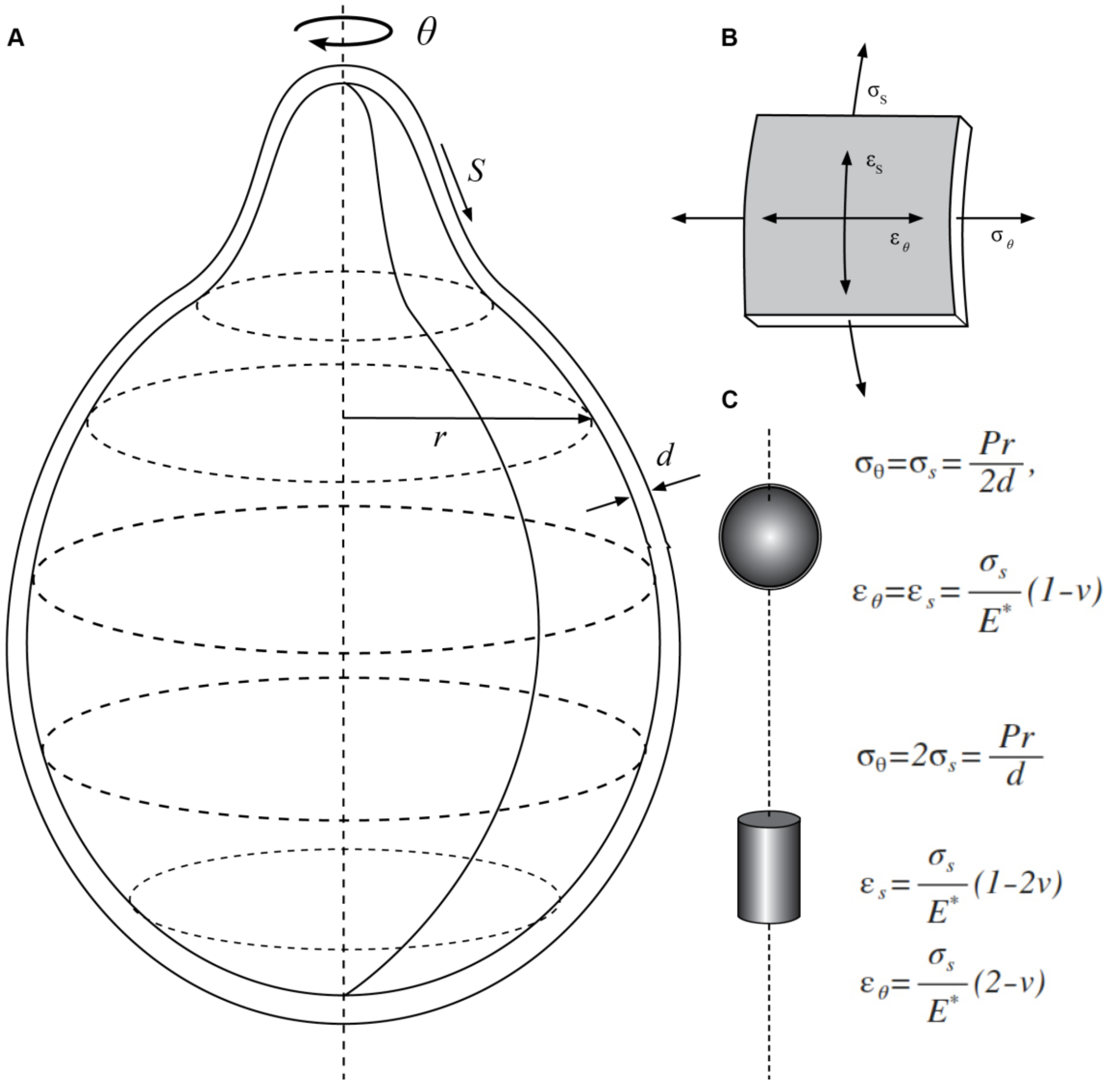

Figure S1. Geometrical considerations and principal in plane stresses and strains. A shows the used coordinates: circumferential angle θ, meridional distance s, radius r and shell thickness d. **B** shows principal stresses and strains for a given shell element. **C** Circumferential and meridional stresses (σ_θ, σ_s) and strains (ε_θ, ε_s) are equal for a sphere, while σ_θ is twice as high as σ_s for the lateral surface of a cylinder. Additionally, ε_θ exceeds ε_s, given that $\nu \leq 0.5$.

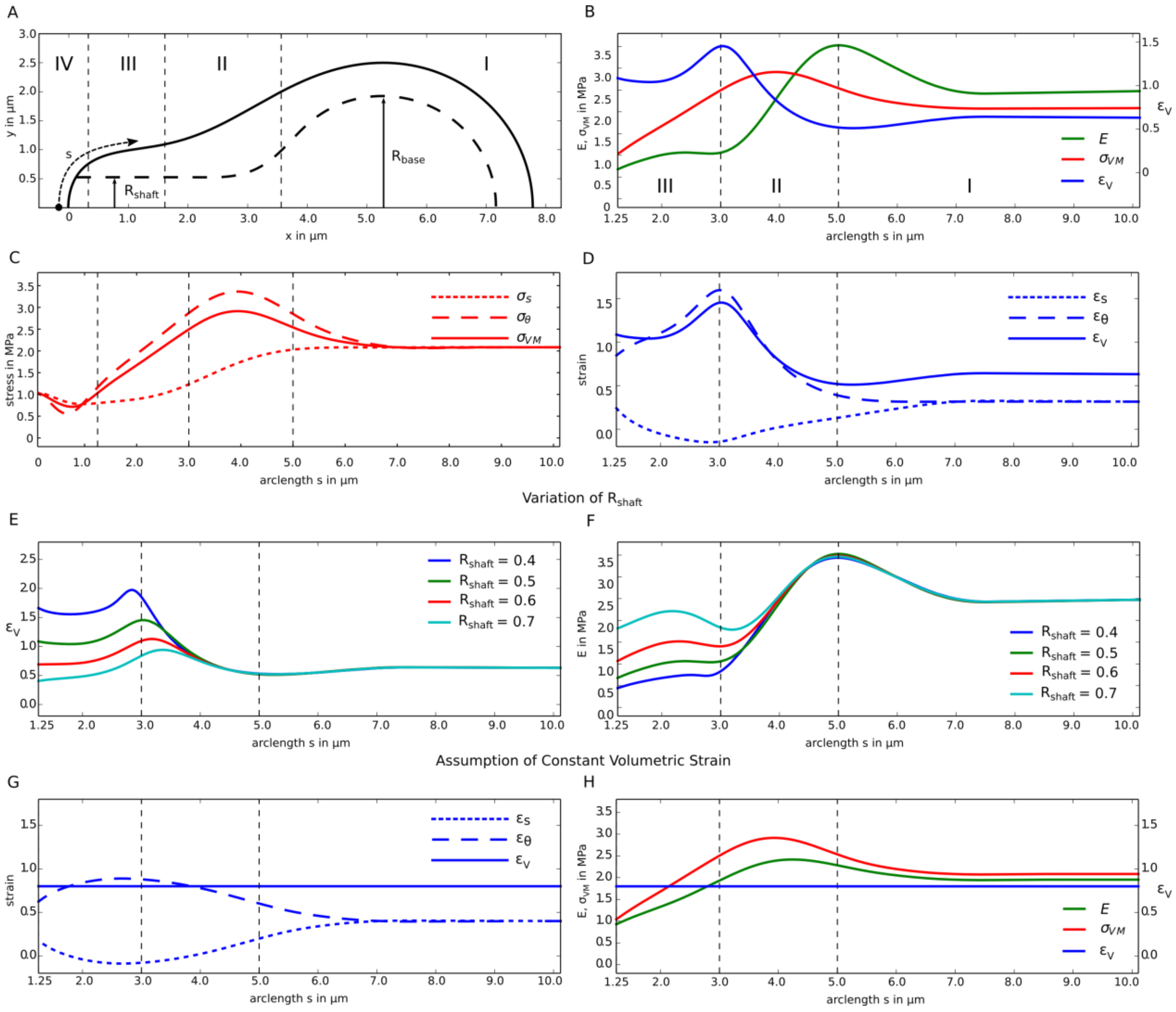

Figure S2. Stress, strain and elasticity profiles of the SM. A Contours for the relaxed (dashed line) and expanded (solid line) cell shape. Tip was the origin of the meridional coordinate, indicated with dashed arrow. The regions tip (I), shaft (II), neck (III) and base (IV) are separated by dashed lines for orientation. For the cell shape in **A** profiles of: **B** Young's modulus *E*, von Mises stress σ_{VM} and volumetric strain ε_{V}, **C** meridional stress σ_s, circumferential stress σ_θ and σ_{VM}, **D** meridional strain ε_s, circumferential strain ε_θ and ε_{V} are shown**. E, F** ε_{V}- and *E*-profiles for different relaxed shapes with varying radius $R_{\mathrm{shaft.}}$ were calculated. **G, H** Profiles of ε_s and ε_θ or *E* and σ_{VM} assuming a constant volumetric strain ε_{V} and the expanded cell shape in **A**.

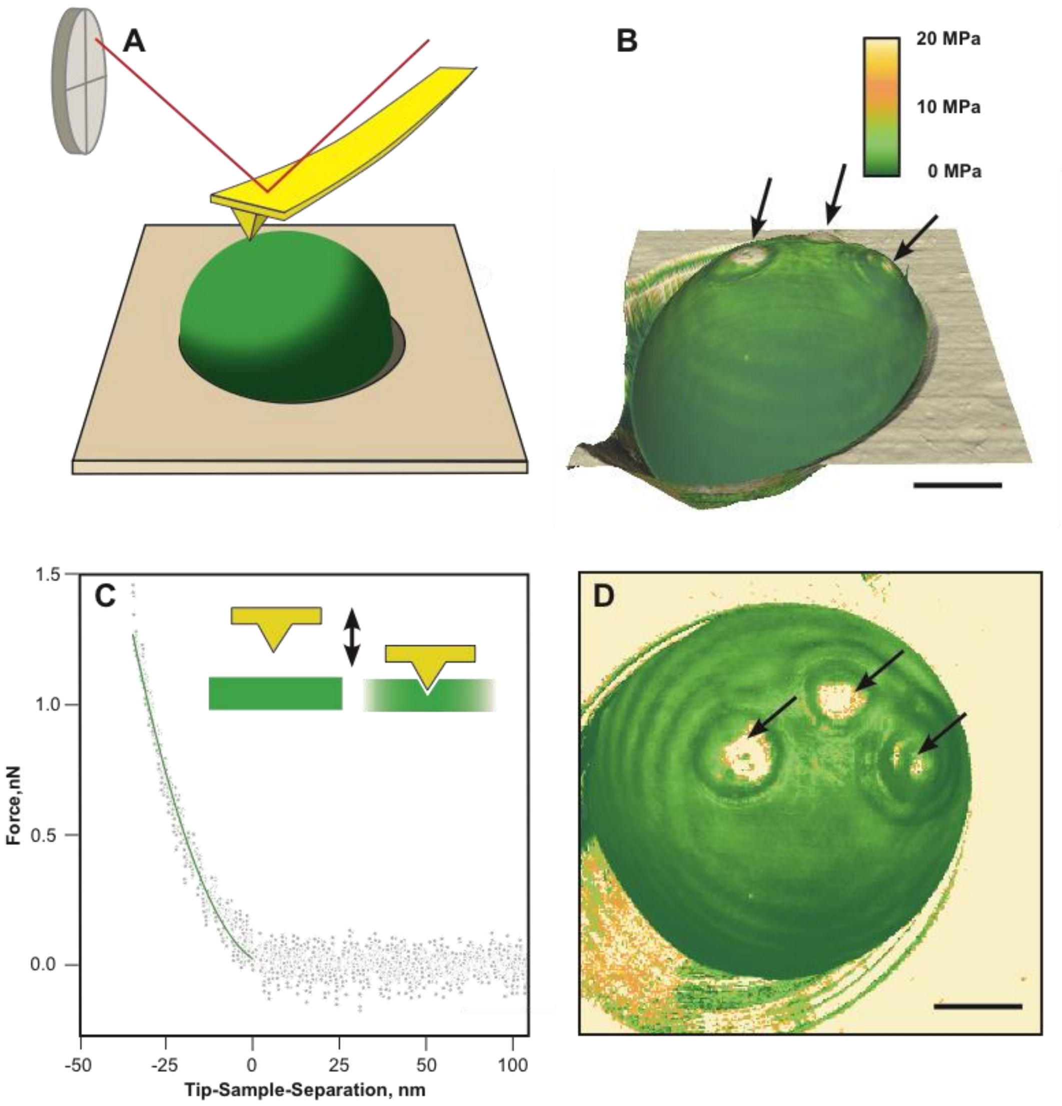

Figure S3. Mapping the cell wall elasticity of entrapped haploid *S. cerevisiae* cells. **A** Scheme of the experimental setup: the previously trapped yeast cell is scanned by an AFM in QI^{TM} Mode. For each pixel a nano-indentation measurement was carried out. **B** 3D representation of the shape detected in **A**. **C** Hypothetic approach curve for one pixel in **A**, along with a Sneddon fit (green) of these data assuming a conical indenter. The resulting spatial information on the elasticity (0 MPa – 20 MPa) of the probed material was displayed in 2D images **D** or used as a texture for 3D representations **B. B** and **D** show the same *MAT**a** bar1*∆ cell with characteristically stiffer bud scar regions (indicated by arrows); scale bar is 1 µm.

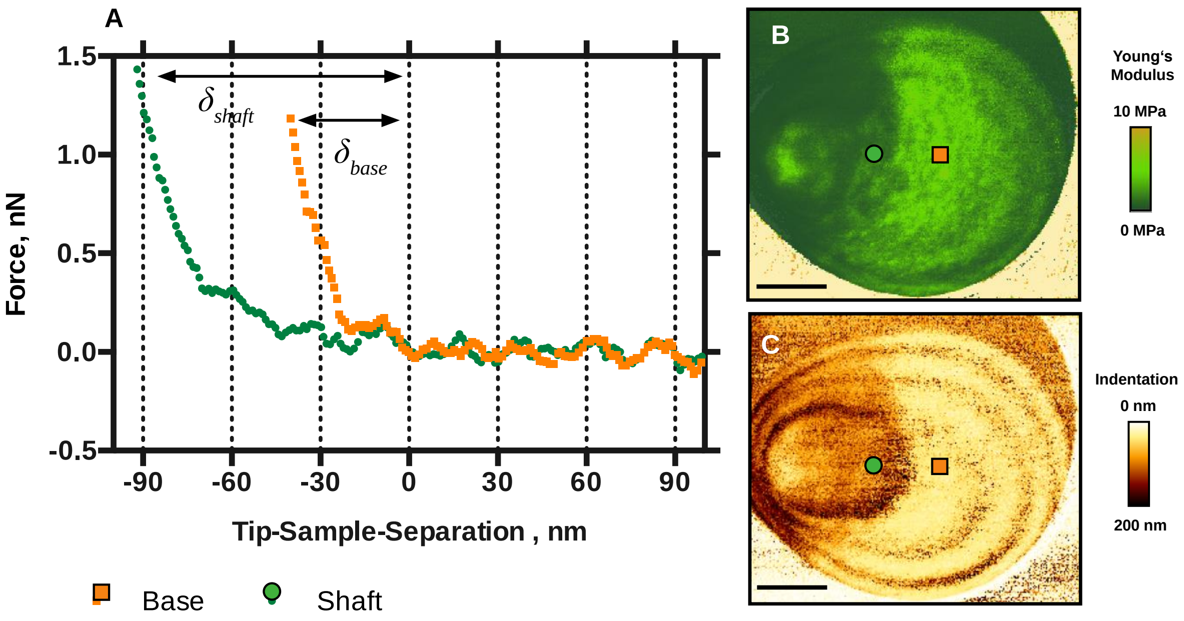

Figure S4. Indentation depth varies between stiffer and softer regions. A Exemplary force-distance curves of nano-indentation measurements at the marked region in **B** and **C**. **B** shows elasticity map and **C** indentation map of shmooing *MAT**a** bar1*Δ cell shown in Figure 2. If both curves reached similar maximum force, the conical tip of the cantilever indented the cell wall in softer region (shaft, green dots) deeper than the stiffer region (base, orange squares), due to the smaller slope of the curve **B**. The indentation depth δ (mean ± Ra, N=900) of a quadratic region (550 nm × 550 nm) at the top of the cell was at the shaft (δ_{shaft} = 97 nm ± 26 nm) and at the base (δ_{base} = 38nm ± 10 nm) less or equal than 115 nm, which is supposed to be the thickness of the cell wall.

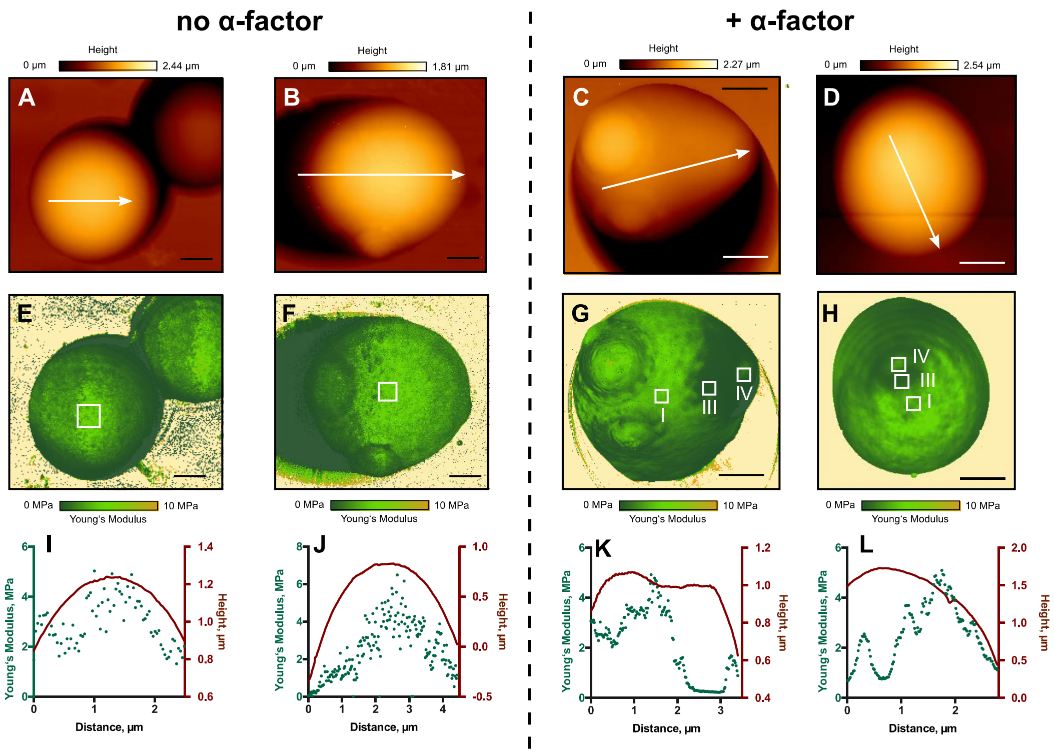

Figure S5. Comparison of height and elasticity patterns between α-factor treated and non-treated *MAT*a *bar1*Δ cells. A, B Height images and **E, F** elasticity maps of two non-treated cells and **C, D** height images and **G, H** elasticity maps of two individual *MAT***a** *bar1*Δ cells treated with 10 µM α -factor; black and white bars correspond to 1µm. The white arrows represent position length and direction of the selected cross-sections in **I, J, K, L.** Manually selected regions for base (I), shaft (II) and tip (IV) are framed with white rectangles.

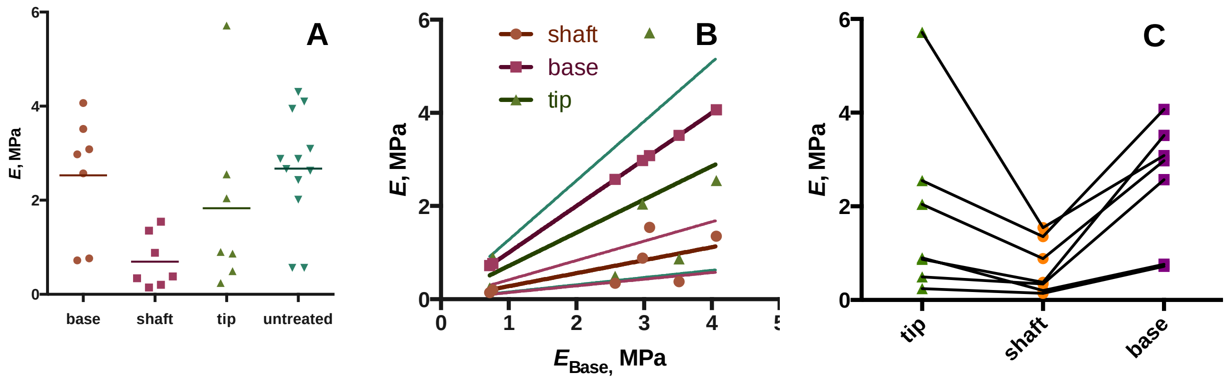

Figure S6. The cell wall, at shaft of the mating projection, showed significant lower *E*-values. **A** Scatter Dot Plot of the mean *E*-values of selected regions at the base, shaft and tip, respectively; bar represents the mean. **B** Mean *E*-values of selected regions at the shaft, the base and the tip plotted against the mean *E*-values at the base for each measured cell. Lines correspond to linear regressions forced through (0,0) with a slope of E_{shaft}/E_{base} = 0.28 ± 0.06 (Sy.x=0.41, DF 6), E_{Tip}/E_{Base}=0.71 ± 0.2 (Sy.x=1.7, DF 6) and E_{Base}/E_{Base}=1.00 ± 0.00 (Sy.x=0.00, DF 6) Broken lines correspond to the respective confidence intervals (95%). **C** Elasticity profiles over tip, shaft and base for all analyzed shmooing cells showed a reduction of *E* from base to shaft and tip to shaft for every cell.

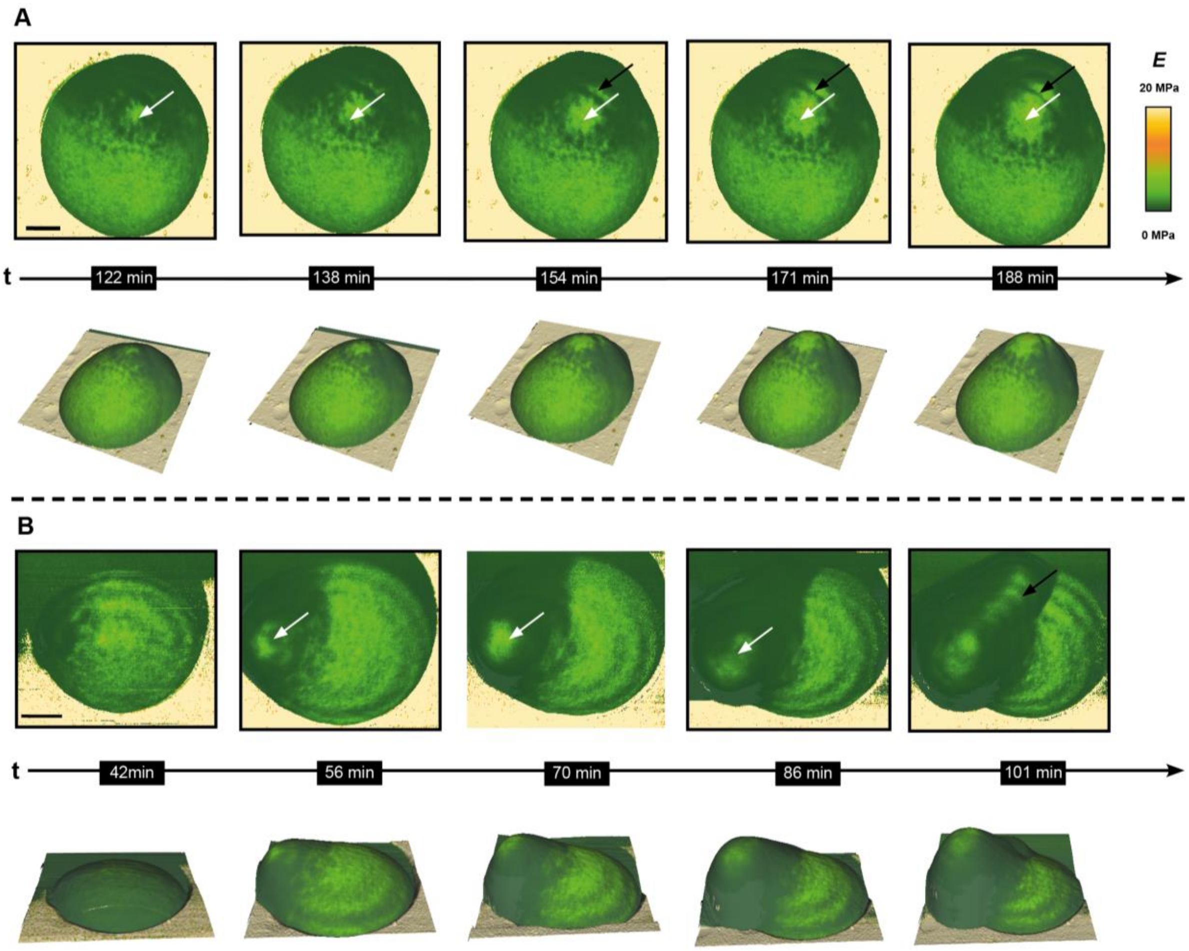

Figure S7. Time series replica for estimation of the cell wall dynamics during mating projection formation. A, B Time-lapse sequence of the height and elasticity development during the formation of a mating projection, obtained with AFM; from left to right, consecutive images of the *E*-distribution (top) and 3D reconstruction with elasticity pattern (bottom), from a continuous measurement. *MAT***a** *bar1*Δ cells were induced with 12 µM **A** and 10 µM **B** α-factor for 122 min **A** and 42 min **B**, respectively, before the first image was acquired. White arrows indicate a region of stiffer cell wall material at the tip. Note, the first image of sequence B shows a barely noticeable reduction in *E* at the side of the emerging protrusion. Both time-laps series show typical AFM-artifacts for high objects (in **A** a doubling of the tip shape and in **B** a height “shadow”), indicated with black arrows.

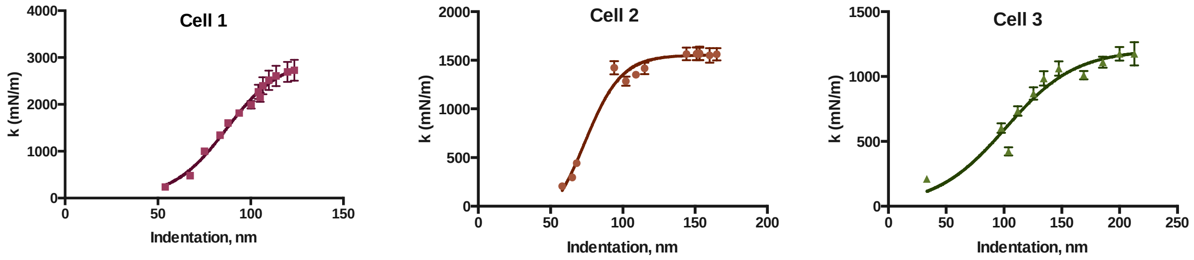

Figure S8. Cell stiffness saturated at 1.5 N/m for high loading forces and large indentations. The cell stiffness, obtained with indentation experiments of three untreated *bar1Δ* cells, was plotted against the loading forces and the corresponding indentation depths. The plotted stiffness *k* (mean, RMS, N=1024), represents the maximal slope of the indentation curve, using a cantilever with *k* of 0.64 N/m. The fitted maximum, assuming a Hill function, was used to calculate the turgor pressure applying the formula: $P = {}^{k}/_{\pi r}$.

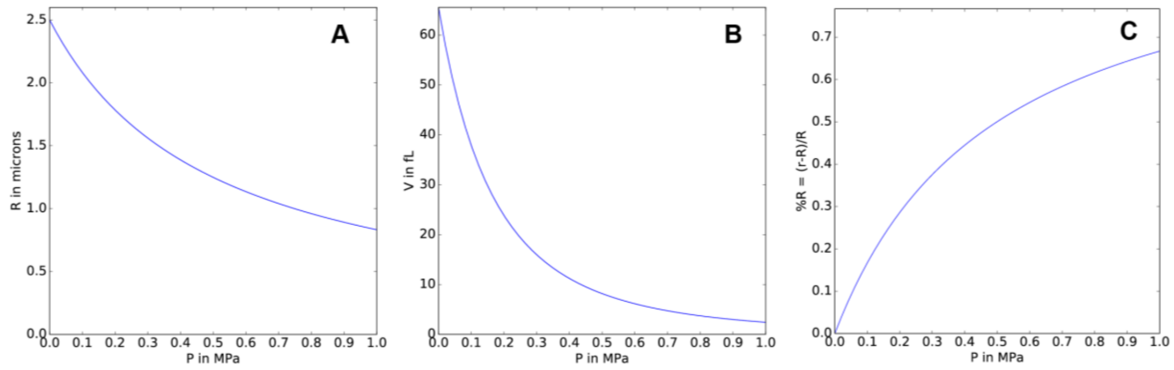

Figure S9. Relaxed shell volume rapidly declines at higher turgor pressure. A shows the relaxed radius of the spherical shell with resting radius 2.5 µm, **B** shows the relative volume confined by the relaxed shell, **C** the relative circumferential strain depending on turgor pressure.

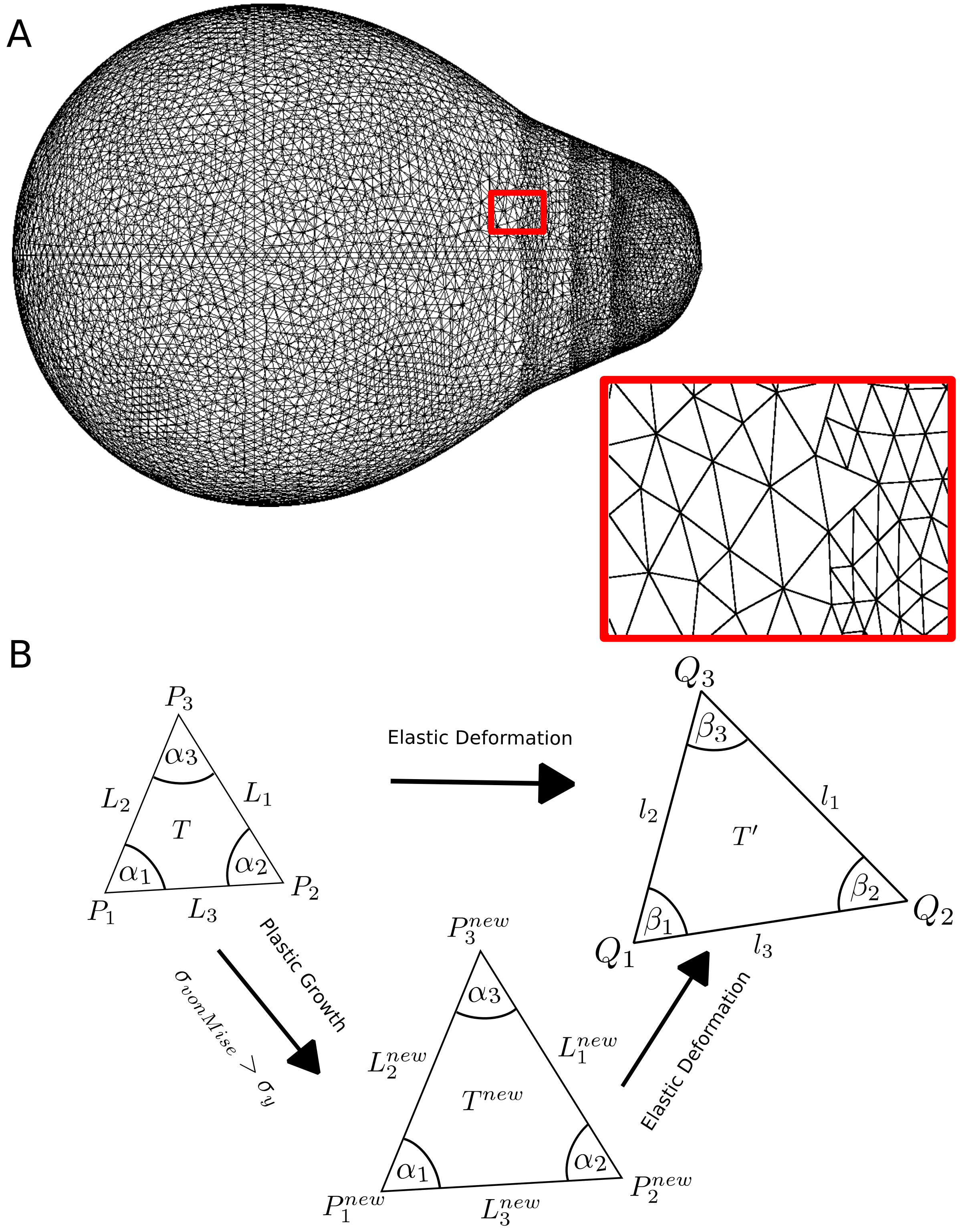

Figure S10. The DM is based on the elasto-plastic deformations of the triangular surface elements. A shows the triangular meshed surface of the simulated shmooing cell, concentric rings at the protrusion results from mesh refinement steps during the simulation. **B** In each simulation step the triangles deform according to the applied stresses. [caption continued, next page]

If the triangle is not in the defined growth zone or $\sigma_{VM} < \sigma_y$, the triangle deforms purely elastically. L_1, L_2, L_3 are the relaxed lengths of the unstressed triangle T and and l_1, l_2, l_3 are the elastically expanded length of the resulting triangle T'. Correspondingly, α_1, α_2, α_3 represent the relaxed angles and β_1, β_2, β_3 the angles of the deformed triangle. Additionally, triangles in the defined growth zone deform plastically if $\sigma_{VM} \geq \sigma_y$. Thereby, the relaxed lengths expand to new relaxed lengths, L_1^{new}, L_2^{new} and L_3^{new}, respectively while the angles remain unaltered.

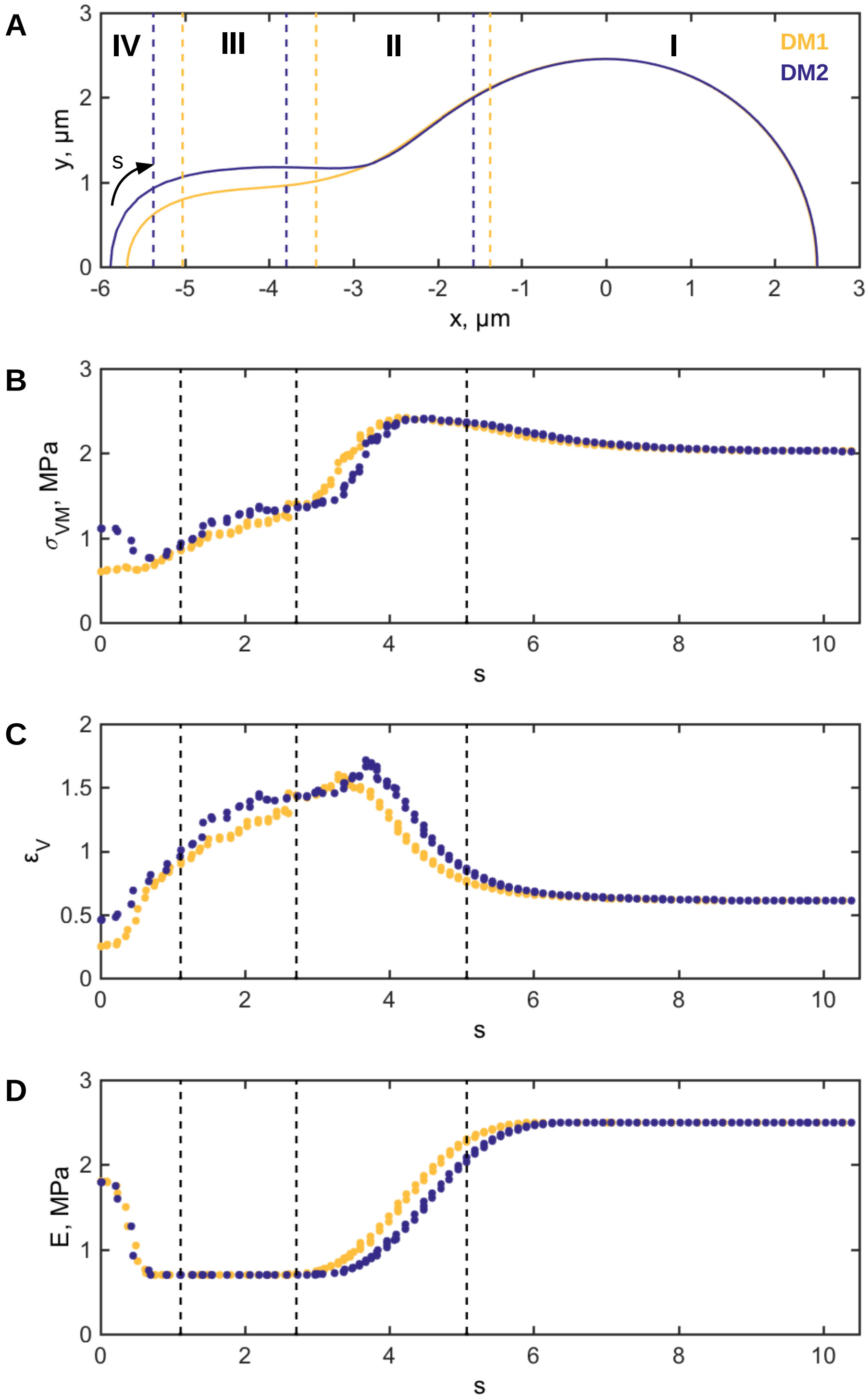

Figure S11. Simulated stress, strain and elasticity profiles along the cell contour. **A** The contours of the resulting cell shape of DM1 (yellow) and DM2 (blue) at time 3250 s with indicated regions: base (I), shaft (II), neck (III) and tip (IV). **B, C, D** Contour plots along the arc length s of the resulting von Mises stress σ_{VM}, the resulting volumetric strain ε_V and the assumed Young's modulus E.

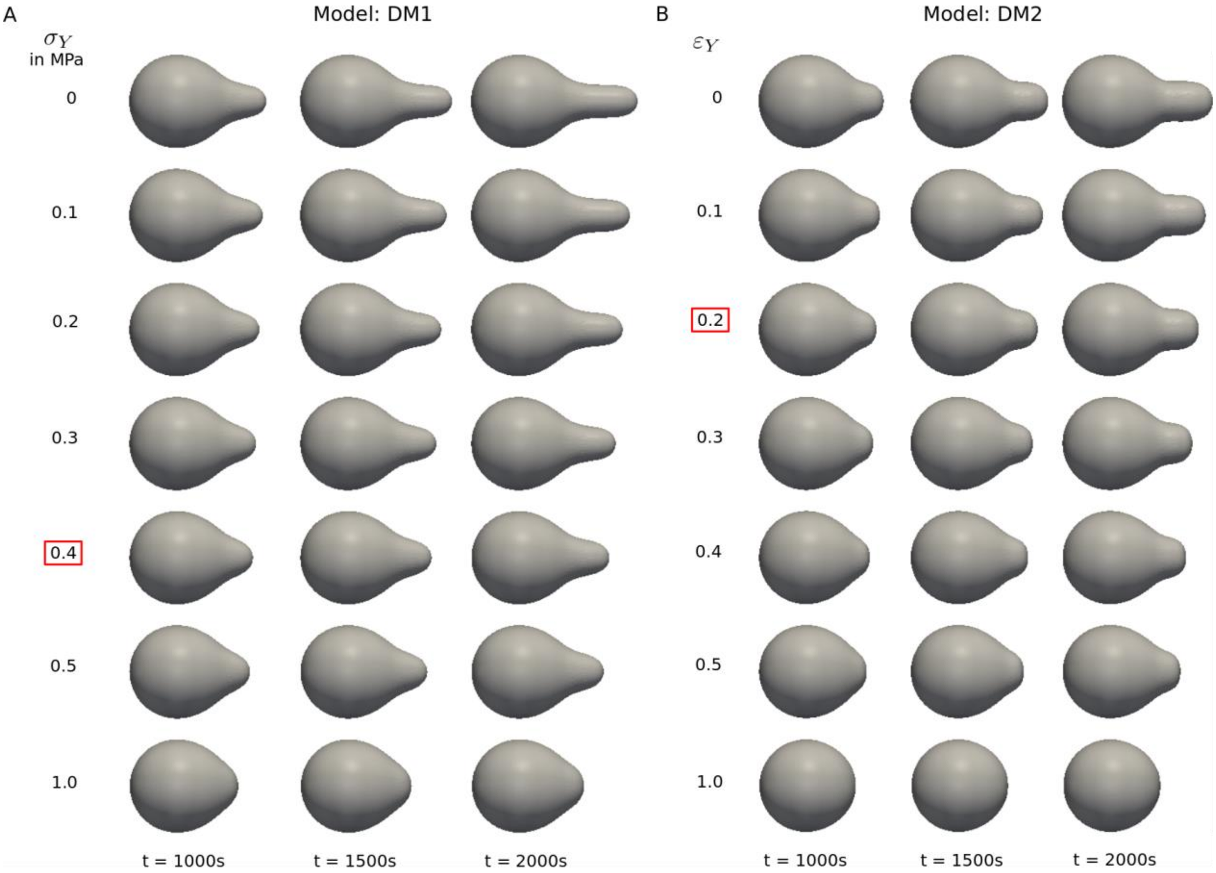

Figure S12. Sensitivity analysis of the yield limit for DM1 and DM2. A and **B show** cell shapes obtained from simulations with various limits for yield stress σ_Y and yield strain ε_Y, respectively at 1000 s, 1500 s and 2000 s. Lower σ_Y or lower ε_Y result in faster growth. When varying parameters in a certain range, similar shapes are obtained at different times.

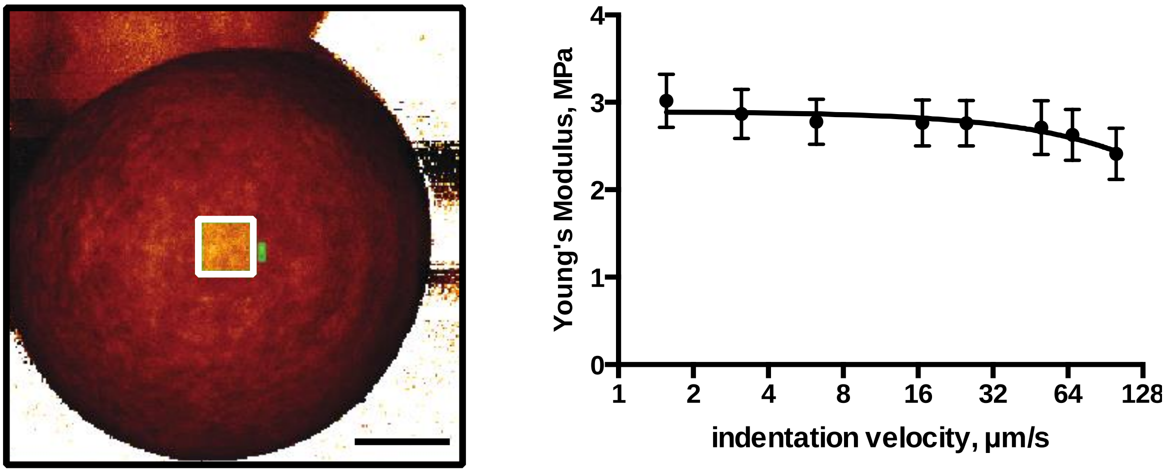

Figure S13. Decrease in Young's modulus with increasing indentation velocity was negligible within the scope of this study (v=67µm/s). The mean Young's modulus of a selected 400nm × 400nm region in the center of a trapped cell (left) plotted against the applied indentation velocity (dots with error bars). Linear regression showed a significant but minor decrease in *E* (slope = -0.0046 ± 0.0008 MPa/(µm/s), R^2=0.85, $F(1, 6)$=33.93 p=0.001).

Table S1 Parameters values used in the models

Property	Abbr.	Value
**General**		
measured turgor pressure	P	0.21 ± 0.05 MPa
cell wall thickness	d	115 nm
Poisson's ratio	ν	0.5
**Steady state model (SM)**		
relaxed base radius	R_{base}	1.9 µm
relaxed shaft radius	R_{shaft}	0.5 µm
expanded base radius	r_{base}	2.5 µm
turgor pressure	P	0.2 Mpa
**Dynamic cell wall models (DM1 & DM2)**		
mass density	ρ	0.5
yield stress	σ_Y	0.4 MPa
yield strain	ε_Y	0.1 MPa
Young's modulus at the base	E_{base}	2.5 Mpa
Young's modulus at the shaft	E_{shaft}	0.7 Mpa
Young's modulus at the tip	E_{tip}	1.8 Mpa
characteristic elasticity time	τ	200s
relaxed base radius	R_{base}	1.9 µm
initial expanded base radius	r_{base}	2.5 µm
radius of enhanced Young's modulus a tthe tip	R_{tip}	0.4 µm
radius of reduced Young's modulus at the shaft	R_{shaft}	0.8 µm
growth radius of the tip	R_{growth}	0.45 µm
extensibility (DM1)	λ	0.004 MPa^{-1} s^{-1}
extensibility (DM2)	λ^*	0.004 s^{-1}
